# Regular afferent activity and structural homogeneity gate ephaptic synchronization in the dorsal column

**DOI:** 10.3389/fnhum.2026.1896515

**Published:** 2026-08-06

**Authors:** Pavel Y. Kondrakhin, Sergei V. Dubrovin, Fedor A. Kolpakov, Boriss Sagalajev

**Affiliations:** Research Center for Genetics and Life Sciences, Sirius University of Science and Technology, Sirius Federal Territory, Sirius, Russia

**Keywords:** computational model, dorsal column, ephaptic coupling, mechanoreceptors, rate coding, spike patterns, temporal coding

## Abstract

Ephaptic coupling can modify spike timing in closely packed myelinated axons, but its effect on physiologically relevant spike patterns remains unexplored. In this study, we used a biophysically detailed model of ephaptic coupling in parallel myelinated axons to analyze how experimentally motivated low-threshold mechanoreceptor spike patterns – regular, semiregular, and irregular – propagate along dorsal column axonal bundles. Spike trains were generated using a statistical procedure and were simulated under different population compositions, with and without ephaptic coupling, and under homogeneous and heterogeneous structural conditions. In our simulations, the homogeneous architecture of the dorsal column preserved the rhythmicity of regular firing inputs. Through ephaptic coupling, this rhythmicity drove synchronization across the entire population. Importantly, this synchronization remained partial and did not escalate into hypersynchrony. Without a regular component, ephaptic coupling alone produced only weak, unstructured activity. Axon diameter influenced propagation speed without altering qualitative dynamics. Also, results were independent of population size. By contrast, diameter heterogeneity abolished rhythmic organization and consequently disrupted synchronization. Semiregular firing – characterized by intermittent spike omissions that produce a bursting structure – consistently lost its rhythmic organization across all scenarios and converged toward irregular firing, which itself exhibited slightly reduced temporal variability during propagation. These results indicate that ephaptic coupling enhances the salience of temporal coding by allowing only fully preserved rhythmic firing to entrain and, thereby, partly synchronize surrounding spikes in the dorsal column. Semiregular and irregular inputs, by contrast, appear to contribute primarily to rate coding.

## 1 Introduction

Low-threshold mechanoreceptors (LTMRs) transmit tactile information from the periphery to the central nervous system and are essential for object recognition, texture discrimination, and the perception of mechanical stimuli (Johansson and Vallbo, 1979; Lumpkin and Caterina, 2007; Abraira and Ginty, 2013). Their axons are large in diameter and heavily myelinated, enabling rapid conduction of tactile signals. In peripheral nerves, LTMR axons are located together with other sensory fibers, including high-threshold mechanoreceptors (HTMRs) with smaller diameters and different myelination properties. Functionally, HTMRs primarily encode nociceptive information, whereas LTMRs are critical for object recognition and tactile discrimination. After entering the spinal cord, however, LTMR afferents ascend through the dorsal column (DC), forming a more structurally homogeneous environment, which may increase their susceptibility to crosstalk mediated by extracellular electric fields (Katz and Schmitt, 1940; Arvanitaki, 1942; Anastassiou et al., 2011; Buzsáki et al., 2012; Anastassiou and Koch, 2015). This crosstalk arises because closely packed myelinated axons share the extracellular space. Action potentials propagating along one axon can perturb the extracellular electric potential around neighboring fibers, thereby influencing their membrane potential dynamics and spike timing.

We refer to this phenomenon as “ephaptic coupling” (Katz and Schmitt, 1940; Arvanitaki, 1942), although the criteria we apply may not be universally shared. Alternative terms include “electric field effects” (Ramon and Moore, 1978; Jefferys, 1995). Previous experimental (Anastassiou et al., 2011; Han et al., 2018, 2020; Rebollo et al., 2021; Subramanian et al., 2022; Striebel et al., 2025) and numerical (Bokil et al., 2001; Stacey et al., 2015; Xu et al., 2018; Shifman and Lewis, 2019; Capllonch-Juan and Sepulveda, 2020; Sheheitli and Jirsa, 2020; Jæger and Tveito, 2026) studies have shown that ephaptic coupling can synchronize spikes propagating along adjacent axons. Schmidt and Knösche (2022) proposed a computational model for analyzing these effects in myelinated axonal bundles and showed that ephaptic coupling facilitates synchronization, whereas structural heterogeneity has a dispersive effect and can counteract this tendency.

During active object exploration, skin-surface interactions generate both sustained indentations and texture-specific vibrations (Lieber and Bensmaia, 2022). Sustained indentation evokes sustained firing in slowly adapting LTMRs, with spike timing varying across receptor types. Experimental recordings by Al-Basha and Prescott (2019) have shown that sustained tactile stimulation can evoke several distinct firing patterns in LTMRs: regular firing with low interspike interval (ISI) variability, irregular firing with broadly variable ISIs, and semiregular firing, in which an approximately regular spike train contains intermittent missing spikes. In contrast, during vibratory stimulation, LTMR activity can become more regular and phase-locked (Handler and Ginty, 2021).

The synchronization of DC activity is a key factor linking axonal spike propagation to sensory perception. Recent work on spinal cord stimulation by Sagalajev et al. (2024) has demonstrated that high-frequency stimulation can activate DC axons without producing paresthesia because spikes become less synchronized, while asynchronous spikes are preferentially blocked by feedforward inhibition and fail to activate somatosensory cortex.

Most modeling studies on ephaptic coupling have focused on the role of structural and morphological parameters of axonal bundles, such as fiber density, extracellular resistivity, myelin sheath thickness, and the spatial arrangement of nodes of Ranvier (Binczak et al., 2001; Bokil et al., 2001; Reutskiy et al., 2003; Ladenbauer and Obermayer, 2019; Schmidt and Knösche, 2019). This approach is essential for understanding the physical conditions under which ephaptic synchronization can occur. However, the diversity of LTMR firing patterns raises an additional important question: how are LTMR spike trains transformed as they propagate along the DC under the influence of ephaptic coupling, and under which structural conditions can their interaction promote temporal organization of the propagated signal?

In our previous conference study (Kondrakhin et al., 2025), we made an initial step toward addressing this question by integrating physiologically motivated LTMR spike patterns (Al-Basha and Prescott, 2019) into a biophysically detailed model of ephaptic coupling (Schmidt and Knösche, 2022). The results indicated that regular firing supports stronger temporal organization of population activity during propagation along the DC. However, that analysis was limited to a narrower set of simulation conditions. To address this limitation, the present study systematically examines how spike-pattern composition, ephaptic coupling, axon diameter, axon number, and structural homogeneity affect the temporal organization of propagating tactile signals. We simulate regular, semiregular, and irregular LTMR spike patterns in homogeneous and heterogeneous axonal bundles, compare mixed pattern compositions with a full-irregular condition, evaluate propagation with and without ephaptic coupling, and test whether the observed pattern-dependent dynamics depend on axon number or diameter. Understanding these interactions is critical for predicting how tactile information is encoded and potentially distorted during spinal cord transmission.

## 2 Methods

### 2.1 Computational model

We used a biophysically detailed model proposed by Schmidt and Knösche (2022) to simulate ephaptic interactions between parallel myelinated axons. The model is derived from a multi-axon extension of the classical cable equation (Basser, 1993) and is based on the assumption that action potentials propagating along neighboring axons perturb the extracellular electric potential and thereby influence the membrane potential and conduction velocity of nearby fibers. Each axon is represented as a one-dimensional myelinated cable, and the extracellular space is treated as a shared conductive medium. In this formulation, the membrane potential of the *n*-th axon is defined as the difference between the intracellular and extracellular potentials. The extracellular potential reflects the summed contribution of capacitive, passive, and voltage-gated membrane currents from all axons in the bundle.

For a bundle of *N* axons, the shared extracellular potential is determined by the current balance in the extracellular medium, as described by Equation 1:

$$\sum_{n=1}^{N}C_{m,n}\frac{\partial V_{n}}{\partial t}=-g_{\mathrm{ex}}\frac{\partial^{2}\phi_{e}}{\partial x^{2}}-\sum_{n=1}^{N}(g_{m,n}V_{n}-I_{\mathrm{HH}}(V_{n}))$$ (1)

where $V_{n}$ is the membrane potential of the *n*-th axon, $C_{m,n}$ is the membrane and myelin capacitance of the *n*-th axon, $g_{m,n}$ is the radial membrane and myelin conductance of the *n*-th axon, $g_{\mathrm{ex}}$ is the longitudinal conductance of the extracellular medium, $\phi_{e}$ is the extracellular potential, $I_{\mathrm{HH}}(V_{n})$ represents the voltage-gated ionic currents described by the Hodgkin-Huxley model (Hodgkin and Huxley, 1952) and *N* is the total number of axons in the bundle.

The extracellular potential affects the dynamics of each individual axon. For the *n*-th axon, the intracellular axial current depends on the intracellular potential $\phi_{i,n}$ . Since the membrane potential is defined as $V_{n}=\phi_{i,n}-\phi_{e}$ , the intracellular potential can be written as $\phi_{i,n}=\phi_{e}+V_{n}$ . Therefore, the membrane potential dynamics of the *n*-th axon can be written as Equation 2:

$$\tau_{n}\frac{\partial V_{n}}{\partial t}=\lambda_{n}^{2}\frac{\partial^{2}(V_{n}+\phi_{e})}{\partial x^{2}}-V_{n}+g_{m,n}^{-1}I_{\mathrm{HH}}(V_{n})$$ (2)

where $\tau_{n}$ is the membrane time constant and $\lambda_{n}$ is the electrotonic length constant of the *n*-th axon. This formulation explicitly separates intrinsic axonal propagation, represented by the second spatial derivative of $V_{n}$ , from ephaptic interaction, represented by the second spatial derivative of $\phi_{e}$ . In simulations without ephaptic coupling, the extracellular interaction term $\phi_{e}^{\prime \prime }$ was removed while preserving intrinsic axonal propagation through the $V_{n}^{\prime \prime }$ component of the cable equation.

The contribution of each axon to the shared extracellular potential depends on its diameter. For circular axons, the cross-sectional area is proportional to the square of the axon diameter. Therefore, the curvature of the extracellular potential can be expressed as a diameter-weighted sum of membrane potential second spatial derivatives, as shown in Equation 3:

$$\frac{\partial^{2}\phi_{e}}{\partial x^{2}}=-\kappa \sum_{n=1}^{N}\frac{d_{n}^{2}}{\sum_{k=1}^{N}d_{k}^{2}}\frac{\partial^{2}V_{n}}{\partial x^{2}}$$ (3)

where $d_{n}$ is the diameter of the *n*-th axon and *κ* is defined in Equation 4:

$$\kappa ={\left(1+\frac{\sigma_{\mathrm{ex}}}{\sigma_{\mathrm{ax}}}\frac{1-\rho }{g^{2}\rho }\right)}^{-1}$$ (4)

Here, $\sigma_{\mathrm{ex}}$ and $\sigma_{\mathrm{ax}}$ are the extracellular and intra-axonal conductivities, *ρ* is the fiber density, and *g* is the g-ratio, i.e., the ratio between the axonal diameter and the total fiber diameter, including the myelin sheath. The model was used to simulate spike propagation in homogeneous and heterogeneous axonal bundles. In homogeneous simulations, all axons within the bundle had identical diameters, fixed at either 1 μm or 2 μm in separate simulation sets. In heterogeneous simulations, axon diameters were randomly and independently sampled from a uniform distribution in the range of 2–3 μm.

Homogeneous simulations assumed identical axon diameters across the population, whereas heterogeneous simulations used distributed axon diameters.

Myelinated fibers were represented by alternating nodal and internodal segments. Voltage-gated ionic currents were included only at the nodes of Ranvier, whereas internodal segments were modeled as passive myelinated cable regions. The ionic current term $I_{\mathrm{HH}}$ followed Hodgkin-Huxley formalism (Hodgkin and Huxley, 1952), accounting for sodium and potassium conductances responsible for action potential initiation and propagation.

Spike propagation dynamics were obtained by direct numerical integration of the membrane potential equations along the axons. After spatial discretization of the equations, the resulting system was solved numerically using the explicit Euler method with a time step of dt=0.05 μs. All simulations were run up to a total model time of 1,000 ms. Axon length was set to 1,000 mm to approximate long-range tactile afferent propagation from the periphery toward supraspinal targets. Spike timing was recorded at three positions along each axon: the start point (0 mm), the midpoint (500 mm), and the cranial end (1,000 mm).

### 2.2 Spike pattern generation

The present study extends the previous use of the model proposed in the original study by Schmidt and Knösche (2022) by incorporating physiologically relevant spike patterns of LTMRs. Whereas the original work primarily focused on how morphological parameters, such as fiber density, axon diameter distribution, and extracellular resistivity, influence ephaptic synchronization, here the model was used to analyze how different patterns of afferent activity interact through ephaptic coupling during propagation.

LTMRs can generate different spike patterns depending on the type of tactile stimulation. During sustained static indentation, slowly adapting mechanoreceptors may exhibit irregular or semiregular firing. Meanwhile, during vibratory stimulation, both slowly and rapidly adapting LTMRs generate regular spike trains phase-locked to the periodic stimulus. Therefore, three spike-pattern types were included in the model: regular, semiregular, and irregular firing. These patterns correspond to experimentally observed modes of LTMR activity described by Al-Basha and Prescott (2019).

Following (Al-Basha and Prescott, 2019), spike trains were generated through a statistical description of ISIs using a gamma distribution. The probability density function of the gamma distribution was defined in the standard form shown in Equation 5:

$$f(x;k,\theta )=\frac{1}{\theta^{k}\Gamma (k)}x^{k-1}e^{-x/\theta }$$ (5)

where *x* is ISI, *k* is the shape parameter, *θ* is the scale parameter, and *Γ*(*k*) is the gamma function defined by Equation 6:

$$\Gamma (k)=\int_{0}^{\infty }t^{k-1}e^{-t}\mathrm{dt}$$ (6)

The shape parameter *k* controlled the regularity of the generated spike trains. Irregular spike trains were generated using k=1 , which corresponds to high ISI variability. Regular spike trains were generated using k=500 , resulting in low ISI variability and highly rhythmic firing. To keep the mean ISI constant across different pattern types, the scale parameter *θ* was set inversely proportional to the shape parameter *k*. Thus, changing *k* modified the variability of ISIs without changing the mean firing rate. Semiregular spike trains were generated from initially regular spike trains by randomly removing 30% of spikes. This procedure was used to reproduce the experimentally observed integer-multiple patterning described for semiregular units, in which spikes occur in an approximately rhythmic structure but also contain longer intervals corresponding approximately to integer multiples of mean ISI due to skipped spikes (Al-Basha and Prescott, 2019).

For each axon, spike times were generated and then used as input stimuli for the cable model. Several population-level spike pattern compositions were considered. The physiologically representative scenario included a population in which 64% of axons generated regular spike trains, 18% generated semiregular spike trains, and 18% generated irregular spike trains, matching the experimentally observed proportions of these firing modes in LTMRs. Additional scenarios were considered in which either semiregular or irregular activity was dominant, accounting for 64% of the axonal population, with the remaining two patterns each comprising 18%. This design allowed us to analyze how the prevailing mode of afferent activity affects collective spike propagation dynamics under ephaptic coupling. In addition, a control condition was simulated in which all axons generated irregular spike trains. This allowed us to assess whether the observed changes in the mixed-pattern scenarios arose from ephaptic coupling or from the presence of the regular component.

### 2.3 Visual analysis

**Figure 1:**
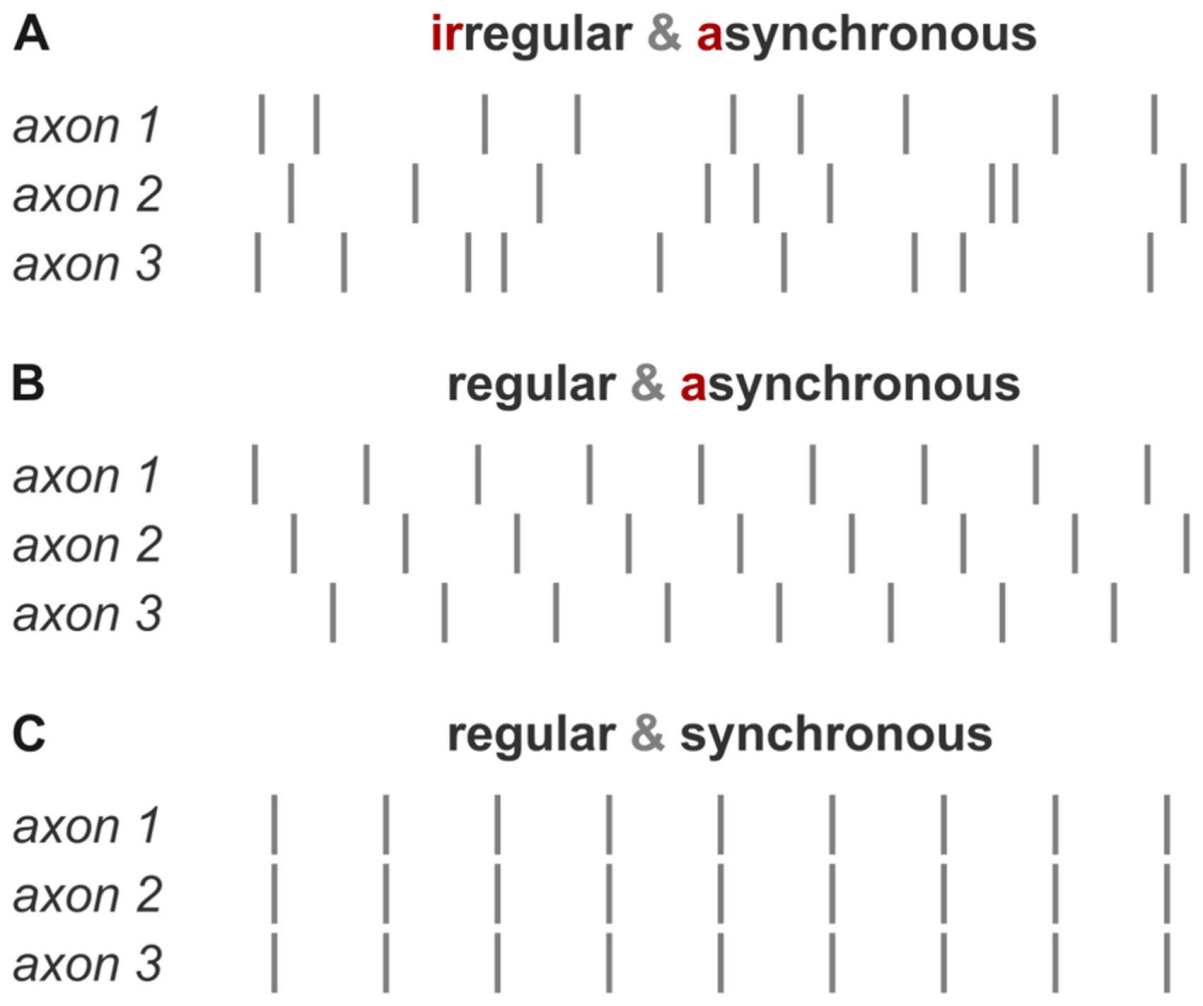
Characterization of firing patterns. **(A)** High ISI variability with poor temporal alignment produces an irregular and asynchronous pattern. **(B)** Reduced ISI variability in the presence of poor temporal alignment yields a regular pattern that remains asynchronous. **(C)** Only a combination of low ISI variability and high temporal alignment produces a pattern that is both regular and synchronous. Vertical markers represent individual action potentials (spikes).

To establish the conceptual framework for interpreting the simulation results, Figure 1 illustrates the distinction between single-fiber regularity and population-level synchrony. Regular firing can occur without temporal alignment between axons, whereas synchronous activity requires both low ISI variability and coordinated spike timing across the population. To assess these distinct phenomena, we structured our analysis as follows:

- Spike regularity was examined using ISI-versus-time plots constructed by calculating ISIs for each axon as the time differences between consecutive spikes, $\text{IS}I_{i}=t_{i}-t_{i-1}$ , where $t_{i}$ is the time of the *i*-th spike at the corresponding recording position. Each ISI value was plotted at the time of the second spike of the corresponding spike pair ( $t_{i}$ ). In addition, normalized ISI density plots were constructed to show how ISI variability differed across spike-pattern types independently of absolute spike count.
- Synchronous activity was examined using spike histograms, which counted spikes across all axons in 1-ms time windows.
- Rhythmic organization was examined using phase plots, in which spike times were converted into phases relative to a fixed reference period (15 ms). Angular distributions were analyzed separately for each spike-pattern type to assess whether their rhythmic organization was preserved during propagation along the axonal bundle. Additionally, angular distributions were pooled across all spike-pattern types to assess changes over time in rhythmic organization of the entire population.

### 2.4 Quantitative metrics

For quantitative support of the visual analysis, ISI variability was quantified using the mean and standard deviation (SD) of ISIs calculated separately for each spike-pattern type, scenario, and recording position. Population-level temporal clustering was quantified from the spike histograms using the coefficient of variation (CV) of binned spike counts. Spike counts were calculated in 1 ms bins, matching the visualization. Bins with zero spikes were excluded to avoid bias from silent intervals, and bins above the 95th percentile of non-zero spike counts were excluded to reduce the influence of isolated extreme peaks. The coefficient of variation was then calculated as the standard deviation of the remaining bin counts divided by their mean. Higher CV spike count values indicate stronger temporal clustering of spikes across the axonal population.

Rhythmic organization was quantified using phase concentration calculated for each phase distribution. For each spike-pattern type, scenario, and recording position, spike phases $\theta_{j}$ were calculated relative to the selected reference period. Phase concentration was calculated according to Equation 7:

$$R=|\frac{1}{n}\sum_{j=1}^{n}e^{i\theta_{j}}|$$ (7)

where R is the phase concentration, n is the total number of spikes included in the phase distribution, $\theta_{j}$ is the phase of the *j*-th spike, *i* is the imaginary unit, and ∣·∣ denotes the magnitude of the complex mean resultant vector. This value ranges from 0 to 1, with higher values indicating stronger concentration of spike phases around a preferred phase.

## 3 Results

### 3.1 Simulation of homogeneous environment

#### 3.1.1 Baseline

The baseline simulations were used as the reference condition for evaluating spike-pattern dynamics in a homogeneous axonal bundle. The bundle consisted of N=100 myelinated axons with identical diameters of d=1 μm, with ephaptic coupling enabled. Three mixed spike-pattern scenarios were analyzed: regular-dominant, irregular-dominant, and semiregular-dominant. In each case, the dominant pattern accounted for 64% of the axonal population, whereas the two remaining patterns accounted for 18% each. This configuration was used to evaluate how the prevailing temporal structure of afferent activity affects ephaptic synchronization under identical structural conditions.

**Figure 2:**
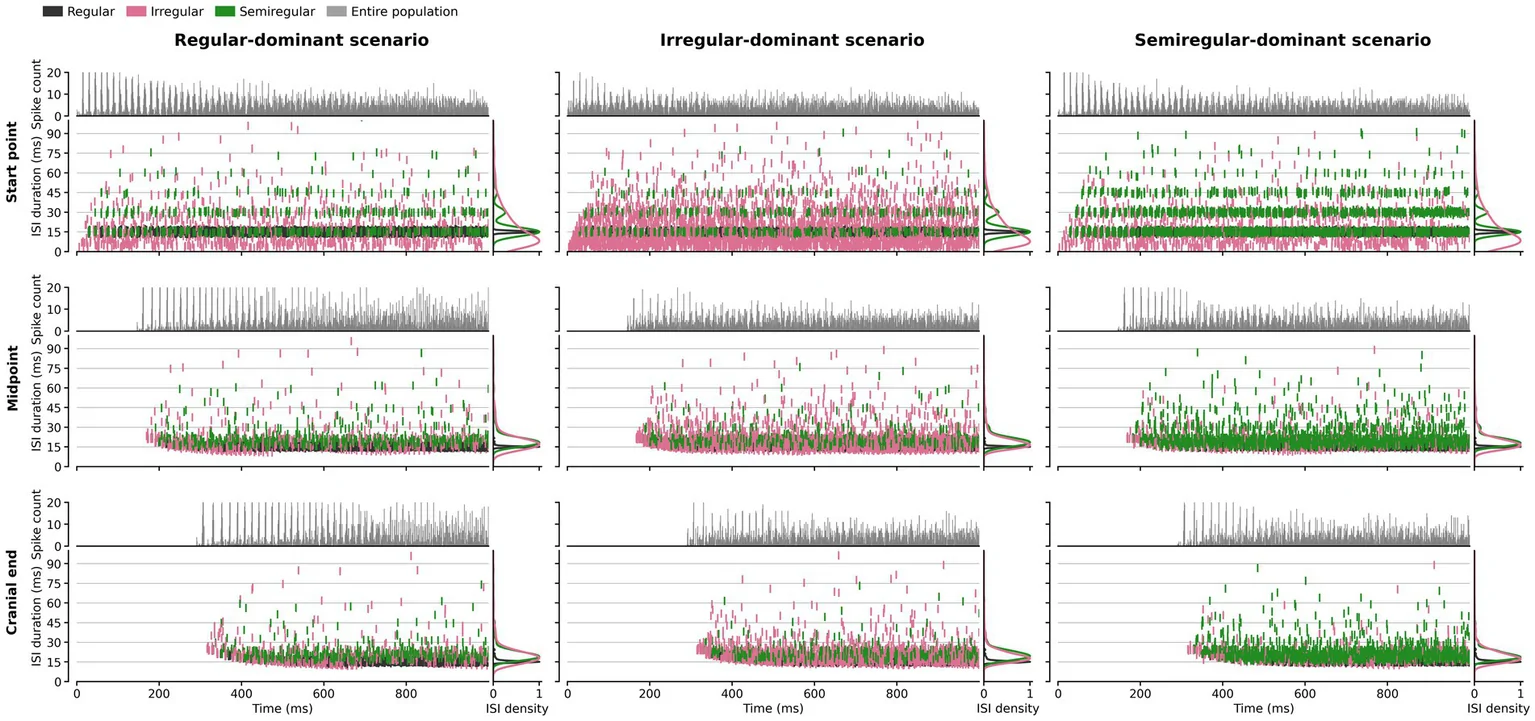
Spike propagation dynamics under baseline homogeneous conditions ( N=100 , d=1 μm). In each panel, the center scatter plot shows ISIs as a function of time for individual axons, revealing pattern-dependent changes in temporal organization during propagation. Each vertical marker on the scatter plot represents the interval between two consecutive spikes of the same axon and is plotted at the time of the second spike of that interval. The line plots on the right display the normalized ISI distribution for each spike-pattern type, enabling comparison of pattern-specific distribution shifts independent of absolute spike count. The grey histogram on the top presents the number of spikes per 1 ms time bin, indicating the level of temporal synchronization across the entire axonal population. The start point reflects the initially generated spike-pattern structure, whereas the midpoint and cranial end capture propagation-dependent transformations induced by ephaptic coupling. The initially high synchronization at the start point reflects the spike-train generation procedure. Because spike times were generated sequentially from stochastic ISIs, timing jitter accumulated gradually across spikes, causing initially aligned spike trains to become progressively desynchronized over time.

Figure 2 scatter plots (ISI duration x time) show the propagation dynamics of regular, irregular, and semiregular spike patterns along the homogenous axonal bundle ( N=100 , d=1 μm). At the start point, the three pattern types retained the distributions imposed during spike generation. The regular firing was narrowly confined to a specific ISI range. The semiregular firing featured the same dominant ISI as the regular pattern but included occasional long ISIs resulting from skipped spikes, which gave it a bursting character. The irregular firing showed the greatest ISI variability, with shorter ISIs predominating. As the spikes propagated toward the midpoint and cranial end, the ISI distributions changed. During propagation, irregular and semiregular firing showed reduced ISI variability, as reflected by a reduction in the standard deviation, whereas regular firing retained a narrow ISI distribution with only moderate broadening (Supplementary Table S1).

Figure 2 histograms (spike count x time) show that spike synchronization depended on the dominant spike pattern. In the regular-dominant scenario, the population exhibited the strongest temporal clustering, visible in the upper spike histograms as more pronounced histogram peaks and supported by the highest CV spike count values at the midpoint and cranial end (Supplementary Table S1). In the irregular-dominant scenario, synchronization was weaker, consistent with the high variability of irregular spike trains. Meanwhile, the semiregular-dominant scenario showed intermediate behavior, yielding only moderate peak formation during propagation.

Figure 2 line plots (ISI duration x ISI density) show that the ISI distributions of the three pattern types grew increasingly similar during propagation (Supplementary Table S1). The regular component remained the most stable, retaining the narrowest ISI distribution throughout. In contrast, the semiregular component lost its bursting structure and transitioned to a more tonic firing, while the irregular component shed its extreme ISIs. Consequently, the latter two distributions became nearly identical, yet remained wider than the regular distribution.

**Figure 3:**
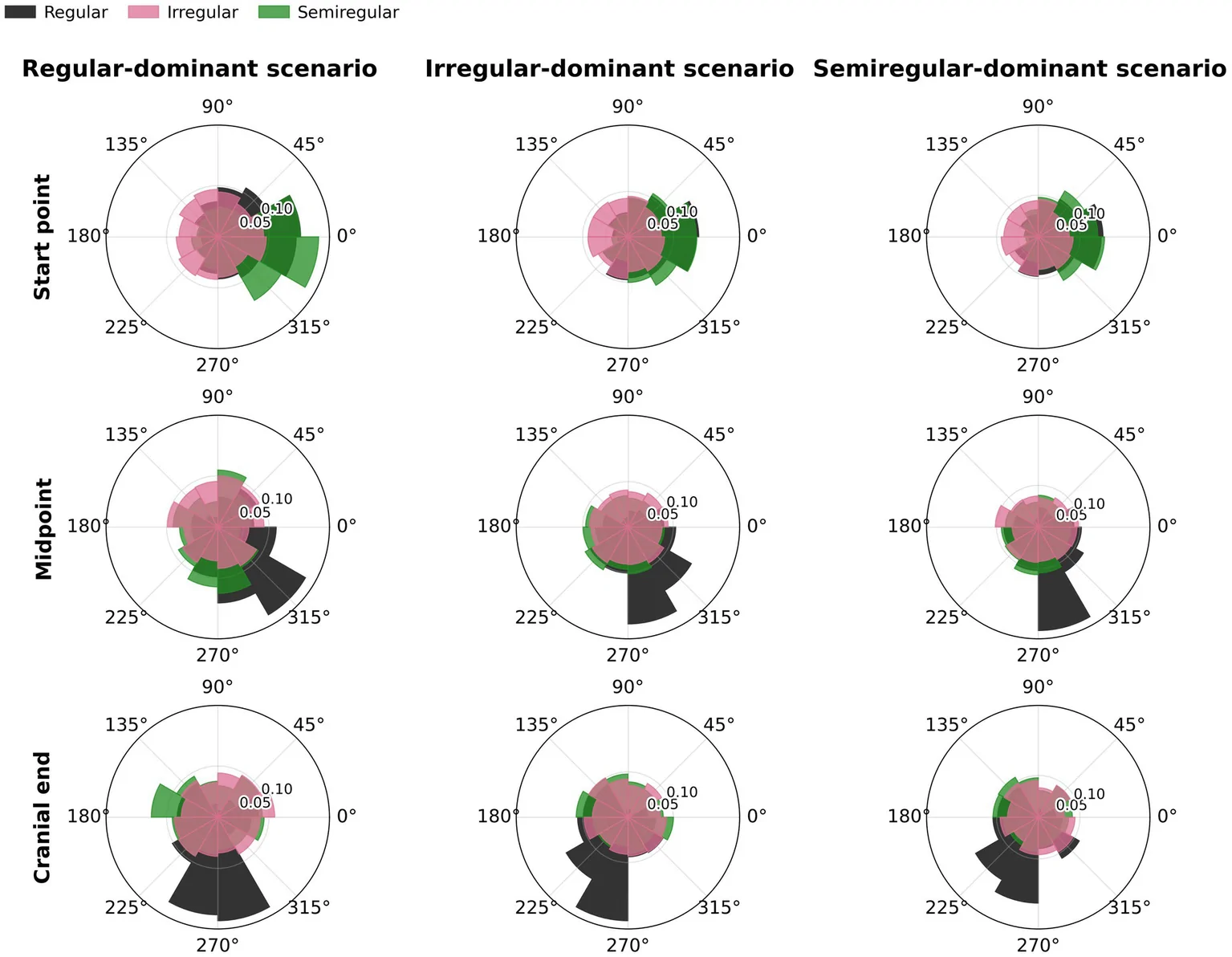
Phase organization of individual spike-pattern types under baseline homogeneous conditions ( N=100 , d=1 μm). Regular firing preserves rhythmic structure most consistently, whereas semiregular firing becomes less phase-concentrated and progressively loses its initial rhythmic organization. Irregular firing remains broadly distributed in phase space, indicating the absence of stable rhythmic organization.

Figure 3 shows phase distributions for individual spike-pattern types under baseline conditions. Regular firing preserved the strongest phase concentration along the axonal bundle, as confirmed by the phase concentration values in Supplementary Table S2, although the preferred phase shifted with propagation. This indicates that regular spike trains maintained their highly rhythmic tonic character. In contrast, semiregular firing showed reduced phase concentration during propagation, indicating that despite transitioning toward a more tonic, regular firing mode, it actually lost some of its initial rhythmicity. Meanwhile, irregular firing remained broadly distributed in phase space.

**Figure 4:**
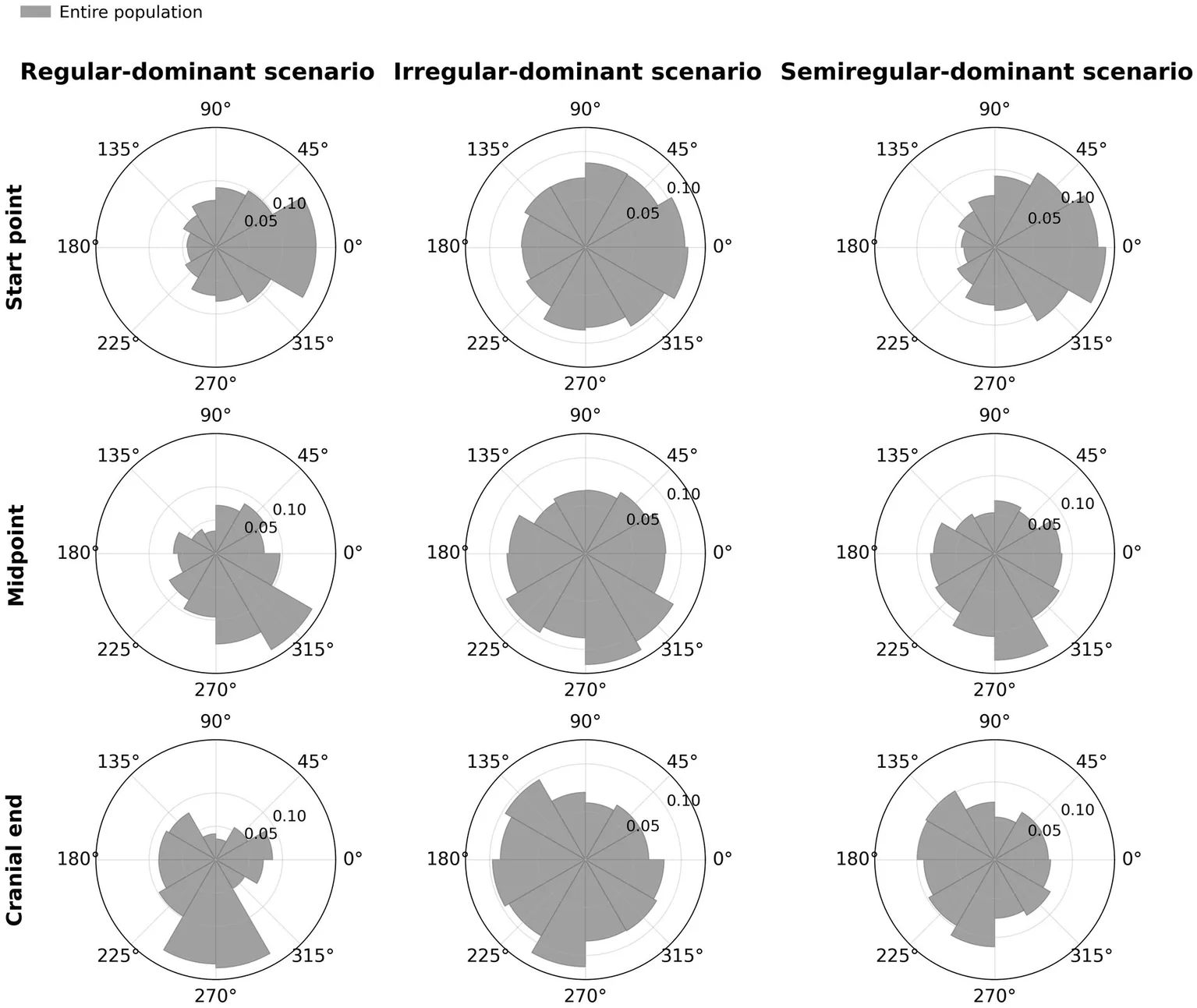
Population-level phase organization under baseline homogeneous conditions ( N=100 , d=1 μm). The regular-dominant scenario shows the strongest global phase concentration, indicating higher population-level rhythmic organization.

Phase distributions in Figure 3 were normalized separately for each spike-pattern type. Therefore, to assess population-level phase organization, we additionally constructed merged phase plots in which all spike-pattern types were pooled together. The results are presented in Figure 4. They confirmed that population-level rhythmicity was strongest in the regular-dominant scenario (Supplementary Table S2). The irregular-dominant and semiregular-dominant scenarios showed broader phase distributions, indicating weaker global rhythmic organization.

Together, these baseline simulations show that, in a homogeneous environment with ephaptic coupling, the presence of a sufficiently rhythmic component not only persists but actively imposes temporal order, driving the synchronization of LTMR spike patterns across the population.

#### 3.1.2 Role of the regular component

To confirm that the temporal order observed in the baseline simulations was imposed by the rhythmicity of the regular component, we simulated a full-irregular scenario in which all axons generated only irregular spike trains, keeping structural parameters identical to baseline ( N=100 , d=1 μm). This condition was compared with the baseline irregular-dominant scenario (64% irregular, 18% regular, 18% semiregular).

**Figure 5:**
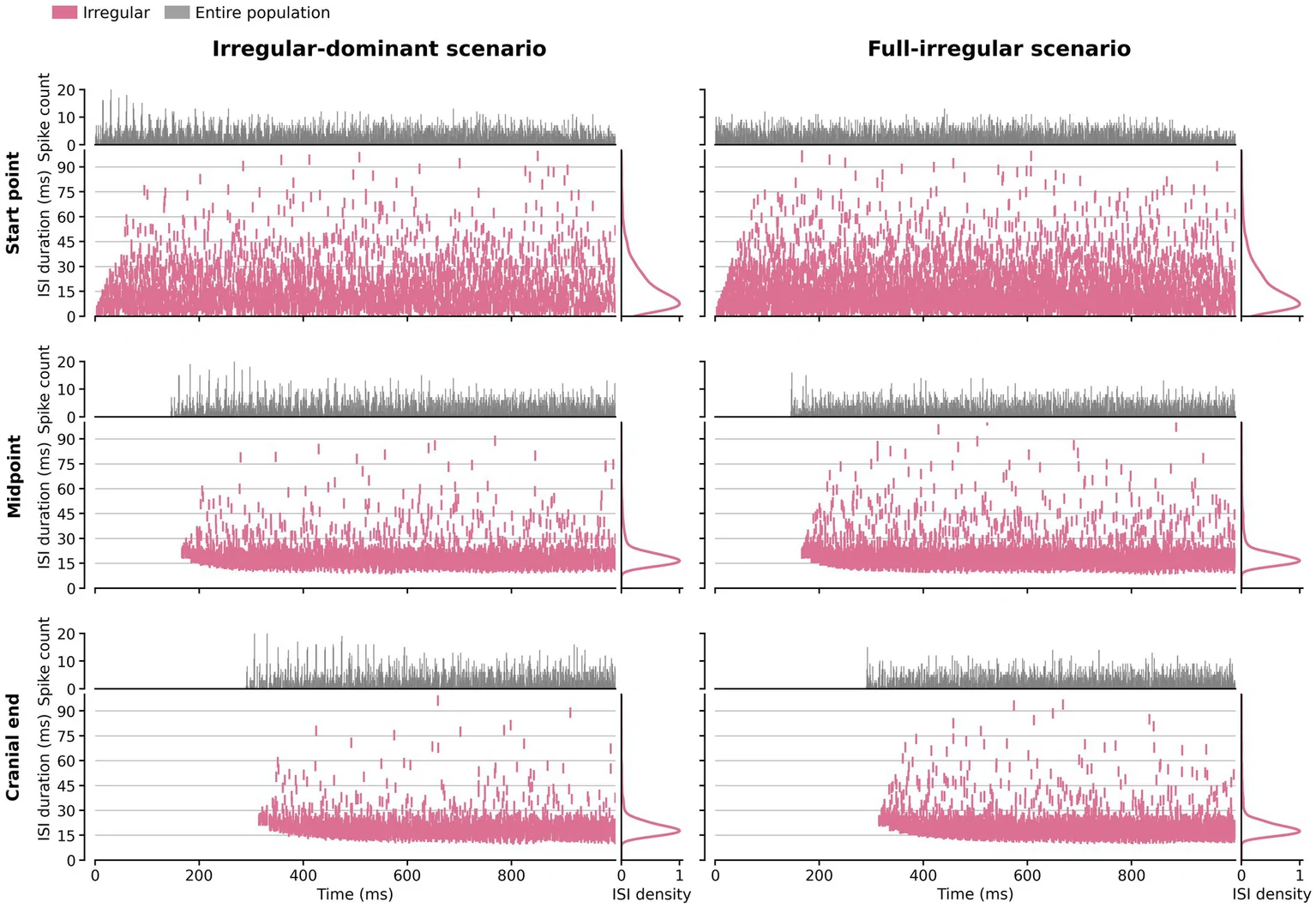
Spike propagation dynamics in the baseline irregular-dominant scenario (left panels) and in the full-irregular scenario (right). Only irregular spikes (vertical markers) are shown in the scatter plots for the left condition, whereas in the right condition all spikes are plotted because the entire population fires irregularly. Structural parameters for the two conditions: N=100 , d=1 μm. In the irregular-dominant scenario, the presence of a regular component (18% of axons) increases population-level temporal synchronization, evidenced by sharper histogram peaks that intensify during propagation. By contrast, in the full-irregular scenario, ephaptic coupling alone fails to generate pronounced temporal organization. ISI scatter and line plots show similar spike distributions between the left and right conditions, characterized by decreased ISI variability during propagation.

The presence of a regular component markedly increased population-level temporal synchronization (Figure 5, left). In the baseline irregular-dominant scenario, the spike histogram showed distinct peaks already at the start point, and the number and sharpness of these peaks increased during propagation toward the midpoint and cranial end (Supplementary Table S1). By contrast, in the full-irregular scenario, spike timing remained weakly organized throughout propagation, with no consistent peak structure emerging (Figure 5, right; Supplementary Table S3). ISI scatter and line plots revealed that the distributions of irregular spikes were largely similar between the two conditions, with each showing reduced ISI variability during propagation.

Thus, these findings confirm that, in a homogeneous environment with ephaptic coupling, the rhythmicity of the regular component is essential for population-wide synchronization of LTMR spike patterns.

#### 3.1.3 Independence on axon number

To test whether the observed spike-pattern dynamics depended on the size of the simulated axonal population, we repeated the baseline simulations using N=1000 axons while keeping the axon diameter unchanged ( d=1 μm). The same three mixed spike-pattern scenarios were analyzed: regular-dominant, irregular-dominant, and semiregular-dominant.

**Figure 6:**
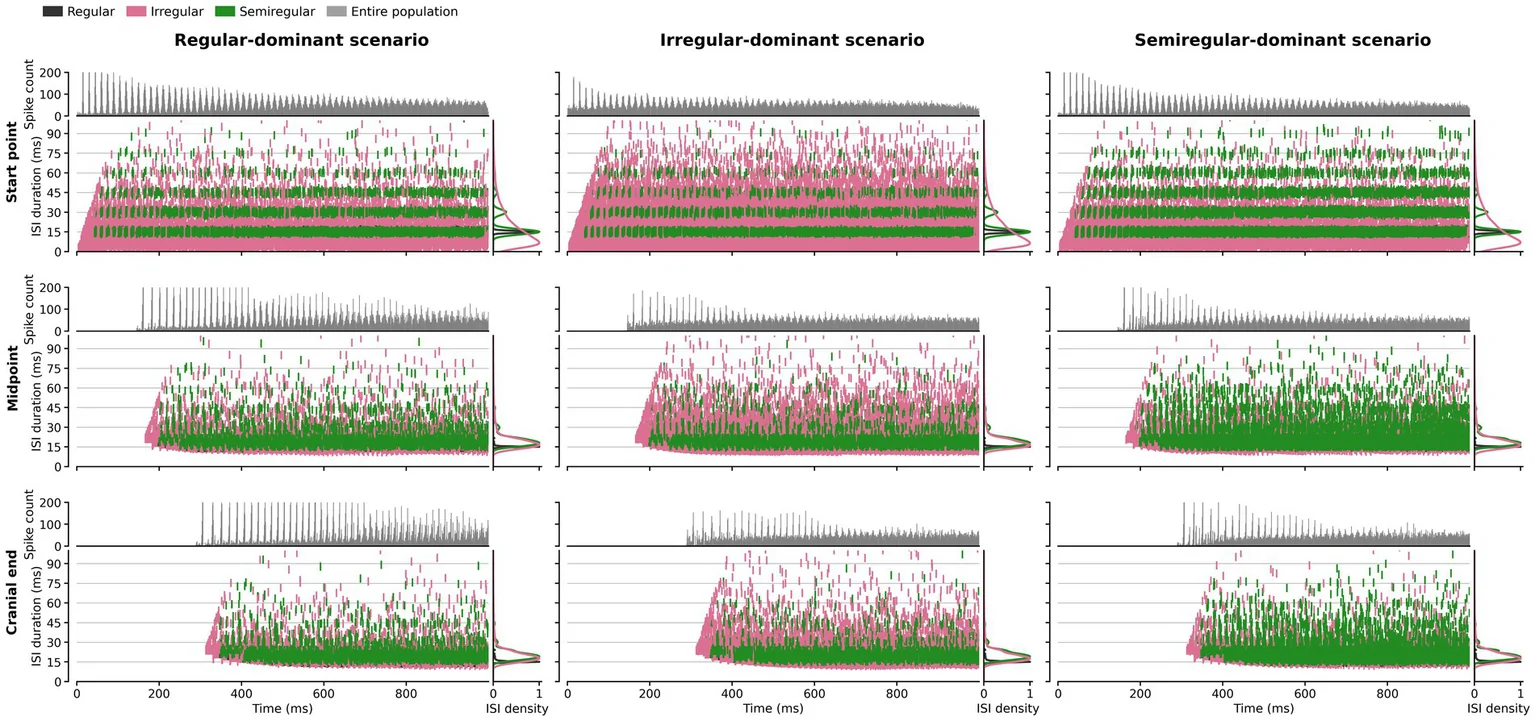
Spike propagation dynamics in a larger population ( N=1000 , d=1 μm). Increasing the number of axons tenfold did not qualitatively change the spike propagation dynamics observed under baseline conditions (see Figure 2). The regular-dominant scenario continued to show the strongest temporal synchronization and maintained the narrowest ISI distribution. Meanwhile, semiregular and irregular firing patterns converged toward similar distributions over the course of propagation.

Increasing the number of axons from N=100 to N=1000 did not qualitatively alter the results observed in the baseline simulations (compare Figure 6 with Figure 2): regular firing remained concentrated within a narrow ISI range, while semiregular and irregular firing showed the same trends toward reduced variability and convergence. The normalized ISI density plots remained qualitatively similar to those obtained for N=100 . The spike histograms also showed the same relationship between dominant spike pattern and synchronization efficiency, with the strongest temporal synchronization observed in the regular-dominant scenario.

These results indicate that the pattern-dependent effects observed in the baseline condition are not specific to the N=100 population size. Increasing the population size mainly increased computational cost without changing the qualitative results. Therefore, subsequent simulations were performed using N=100 axons, which was sufficient to implement the experimentally motivated 64%: 18%: 18% distribution of spike-pattern types (Al-Basha and Prescott, 2019).

#### 3.1.4 Effect of ephaptic coupling

To isolate the contribution of ephaptic interactions to spike synchronization, we repeated the baseline simulations after removing the extracellular interaction term from the membrane potential dynamics. Specifically, the second spatial derivative of the extracellular potential was excluded from the Equation 2 (see Methods), while the intrinsic propagation term was preserved. The simulations were performed using the same structural and population parameters as in the baseline condition: N=100 axons, d=1 μm

**Figure 7:**
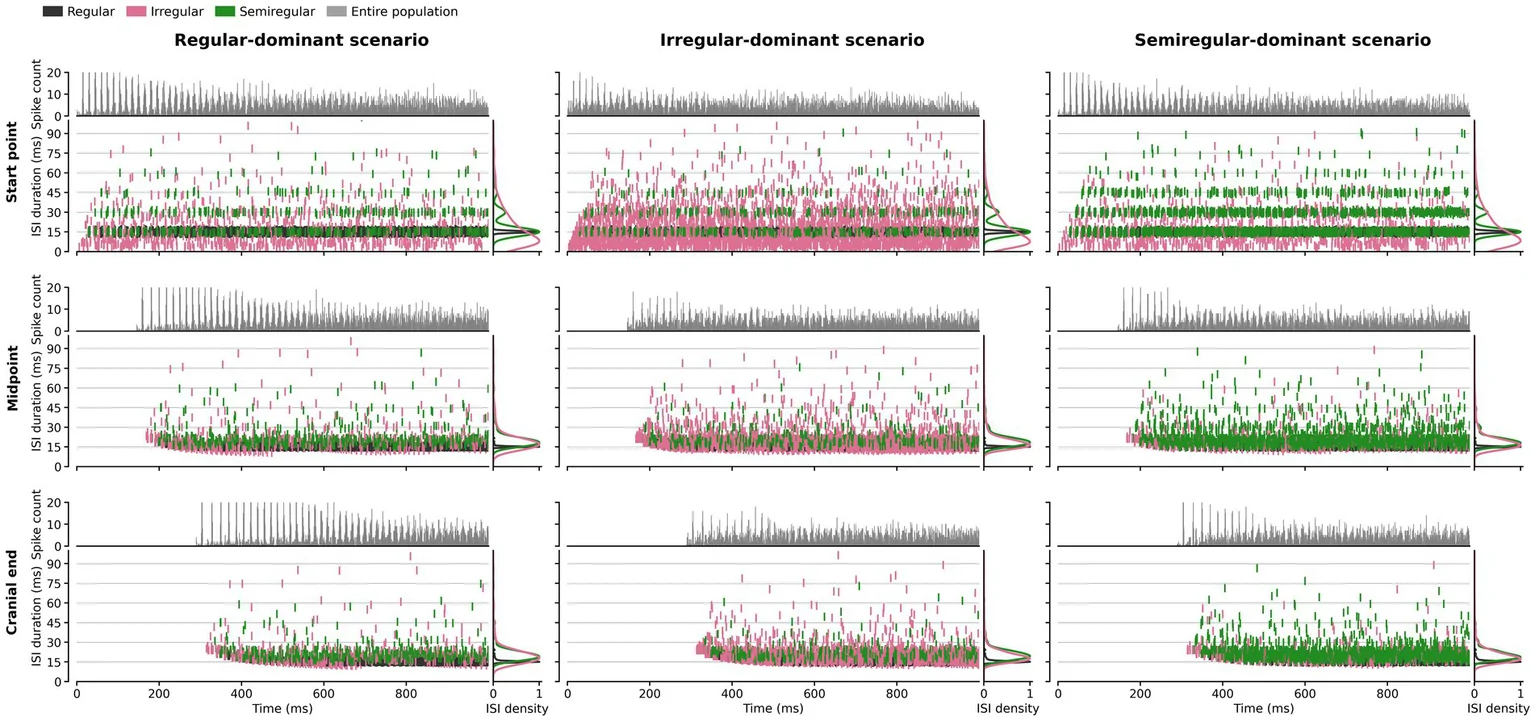
Spike propagation dynamics without ephaptic coupling ( N=100 , d=1 μm). Removal of ephaptic coupling reduced population-level temporal synchronization compared with the baseline condition (see Figure 2), manifested as weaker propagation-dependent peak formation in the histograms. In contrast, the ISI structure remained largely similar to that observed under the coupled condition: emiregular and irregular firing patterns converged during propagation, while regular firing retained a narrow distribution.

Figure 7 shows spike propagation dynamics in the absence of ephaptic coupling. Compared with the baseline simulations shown in Figure 2, the ISI-versus-time scatter plots retained a similar pattern-specific structure. Regular firing remained confined to a narrow ISI range, while semiregular firing gradually lost its bursting organization. Irregular firing showed reduced ISI variability, aligning more closely with the distribution adopted by the semiregular component.

The main difference was observed in the spike histograms. Under baseline conditions with ephaptic coupling, temporal synchronization increased during propagation, as reflected by progressively more pronounced histogram peaks toward the midpoint and cranial end (Figure 2; Supplementary Table S1). However, without ephaptic coupling, these peaks were weaker and showed little to no amplification while propagating along the axonal bundle (Figure 7; Supplementary Table S4).

**Figure 8:**
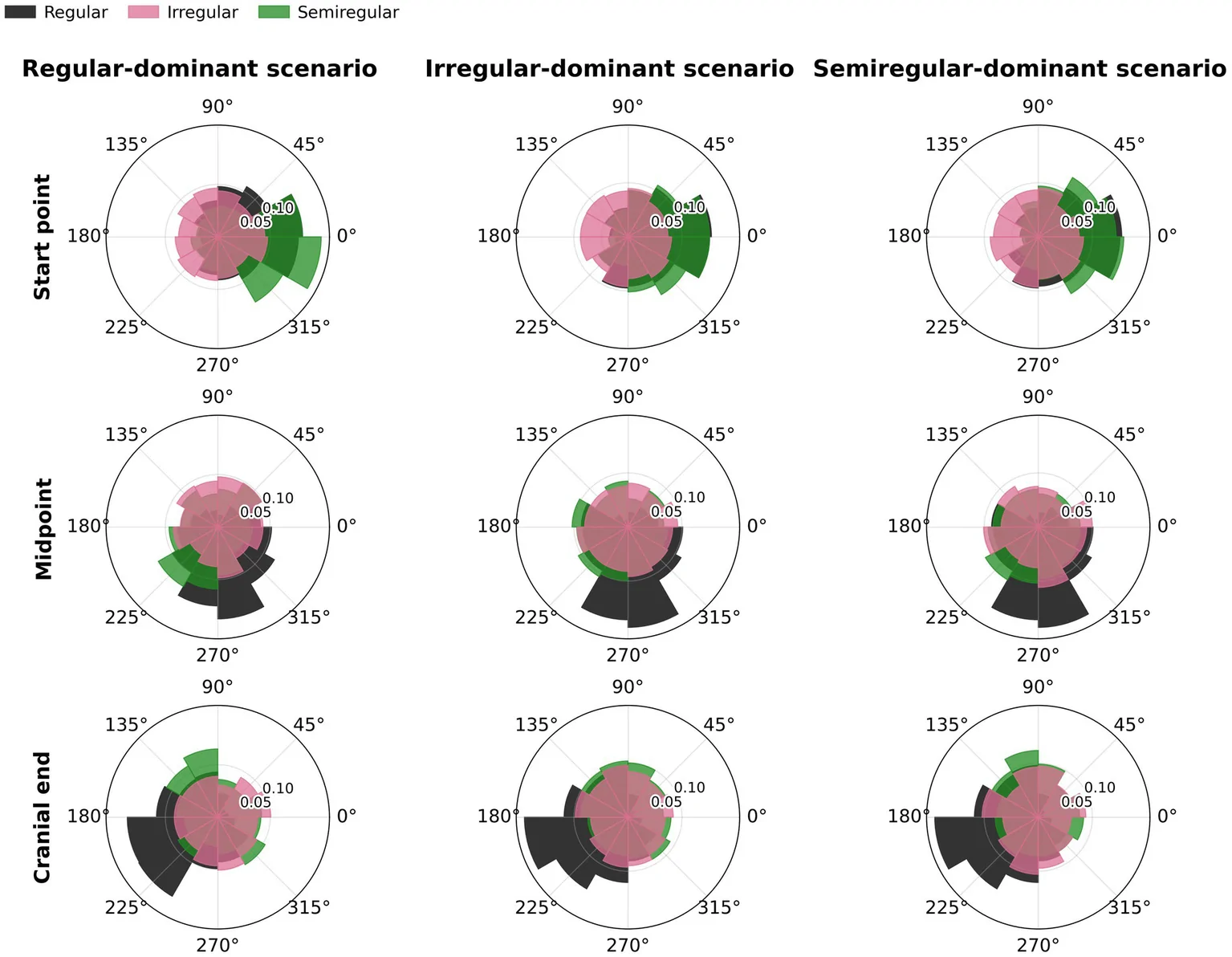
Phase organization of individual spike-pattern types without ephaptic coupling ( N=100 , d=1 μm). Without ephaptic coupling, regular firing retained strong phase organization. In the irregular-dominant and semiregular-dominant scenarios, the propagation-dependent increase in regular phase concentration was slightly weaker than under baseline conditions with ephaptic coupling (see Figure 3; Supplementary Tables S2, S5).

Phase distributions for individual spike-pattern types without ephaptic coupling (Figure 8; Supplementary Table S5) mirrored the baseline (Figure 3; Supplementary Table S2) in that the semiregular component lost its rhythmic structure and the irregular component remained broadly distributed. As in baseline, regular firing retained strong phase concentration. In the irregular- and semiregular-dominant scenarios, however, removal of ephaptic coupling slightly reduced the propagation-dependent increase in phase preference.

**Figure 9:**
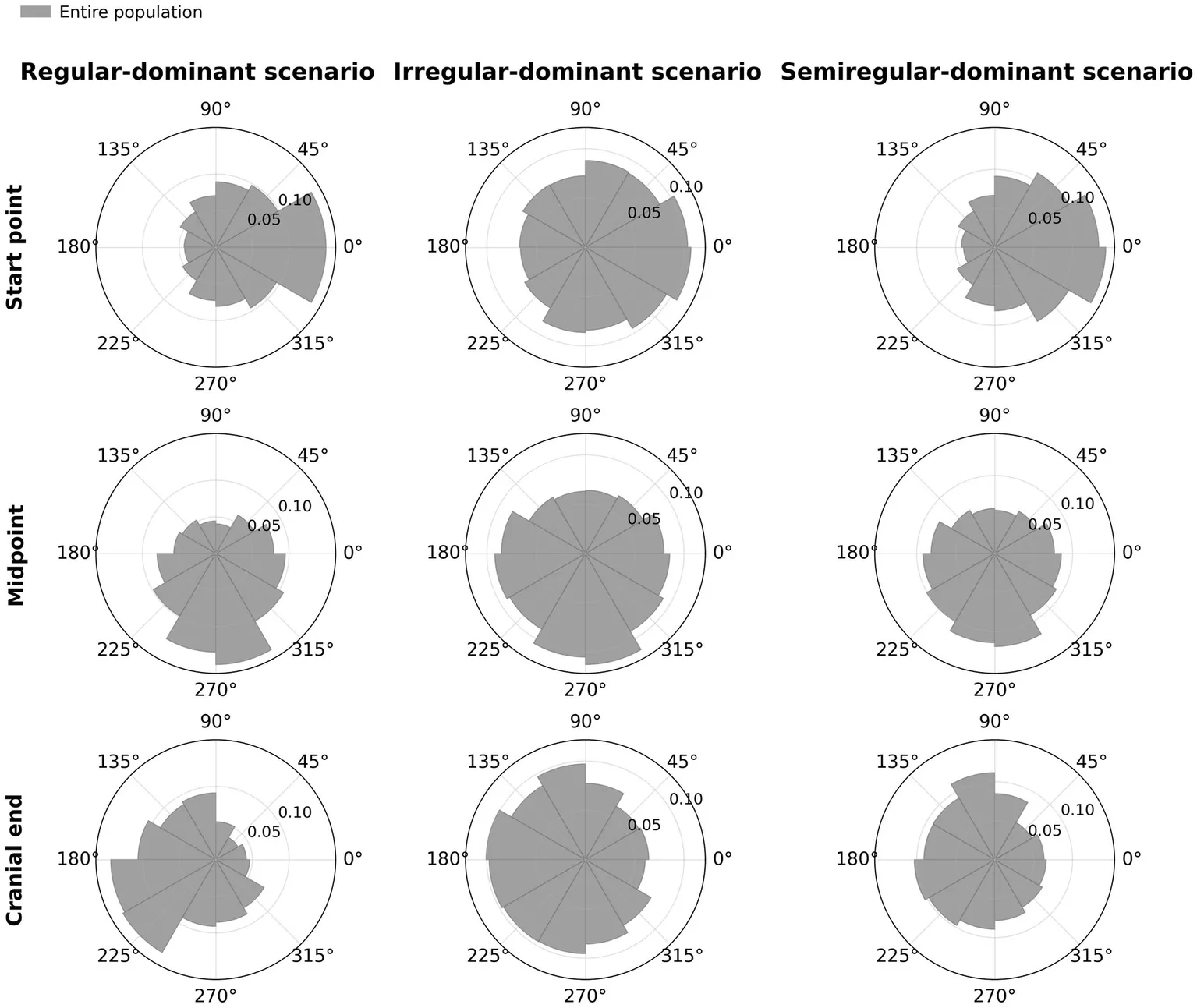
Population-level phase organization without ephaptic coupling ( N=100 , d=1 μm). Merged phase plots show that population-level phase concentration was not abolished by removing ephaptic coupling and remained broadly comparable to the baseline condition (see Figure 4).

At the population level, merged phase distributions showed that removal of ephaptic coupling did not abolish population-level phase structure (Figure 9; Supplementary Table S5), and overall phase concentration remained broadly comparable to the baseline condition (Figure 4; Supplementary Table S2).

Together, these findings show that a homogeneous axonal environment without ephaptic coupling is sufficient to preserve the rhythmic structure of the regular LTMR spike pattern. Ephaptic coupling is therefore required to translate this rhythmicity into population-wide synchronization.

#### 3.1.5 Dependence on axon diameters

To evaluate whether axon diameter affects the spike dynamics observed under otherwise identical homogeneous structural conditions, we repeated the baseline simulations using larger axons. The population size was kept identical to the baseline condition ( N=100 ), whereas the axon diameter was increased from d=1 μm to d=2 μm.

**Figure 10:**
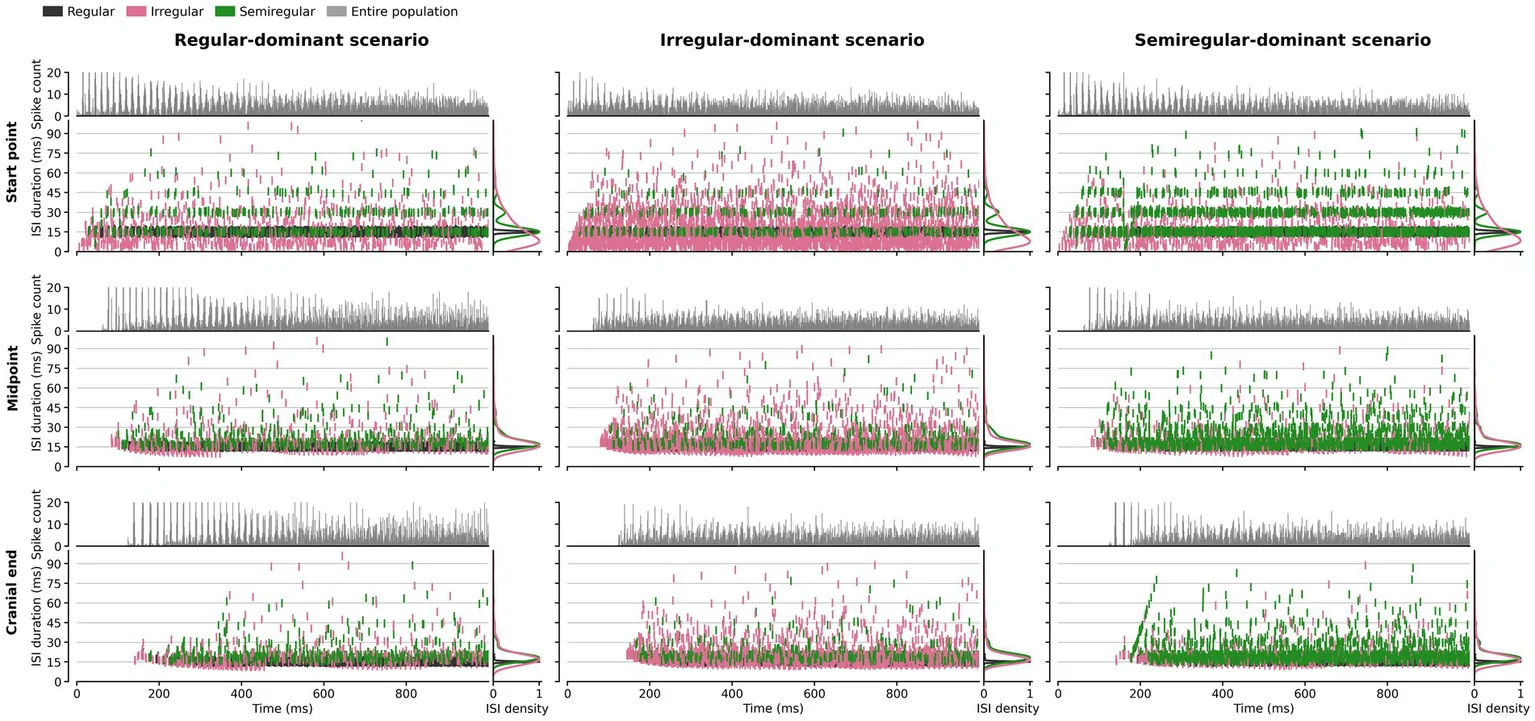
Spike propagation dynamics in larger axons ( N=100 , d=2 μm). Doubling the axon diameter accelerated spike propagation approximately twofold compared with the baseline ( N=100 , d=1 μm), while preserving the qualitative pattern-dependent synchronization dynamics (see Figure 2).

In homogeneous axonal bundles with d=2 μm, spike propagation occurred considerably faster than in the baseline condition with d=1 μm. Under the d=1 μm condition, the first spikes reached the midpoint at approximately 160 ms and the cranial end at approximately 305 ms (Figure 2). Meanwhile, under the d=2 μm condition, the first spikes arrived at the respective points at about 75 ms and 140 ms (Figure 10). Thus, doubling the axon diameter resulted in an approximately twofold increase in conduction velocity, consistent with the expected approximately linear dependence of conduction velocity on axon diameter (Hursh, 1939; Goldman and Albus, 1968).

Despite this change in propagation time, the qualitative organization of spike patterns remained similar to the baseline. Hence, increasing axon diameter primarily affects the speed but not the nature of the observed effects.

### 3.2 Simulation of heterogeneous environment

After analyzing homogeneous axonal bundles, we examined whether structural heterogeneity affects spike rhythmicity and synchronization. The previous homogeneous d=2 μm simulations showed that increasing axon diameter mainly accelerated propagation while preserving the qualitative pattern-dependent dynamics. Therefore, the heterogeneous condition was introduced to test whether variability in axon diameter, rather than larger diameter itself, disrupts temporal organization during propagation.

In contrast to homogeneous simulations, where all axons had the same diameter, the heterogeneous condition included axons with diameters uniformly distributed in the range of 2–3 μm. This configuration was used to approximate a more physiologically realistic axonal population (Duval et al., 2019), while preserving the same number of axons and spike-pattern compositions as in the baseline simulations.

**Figure 11:**
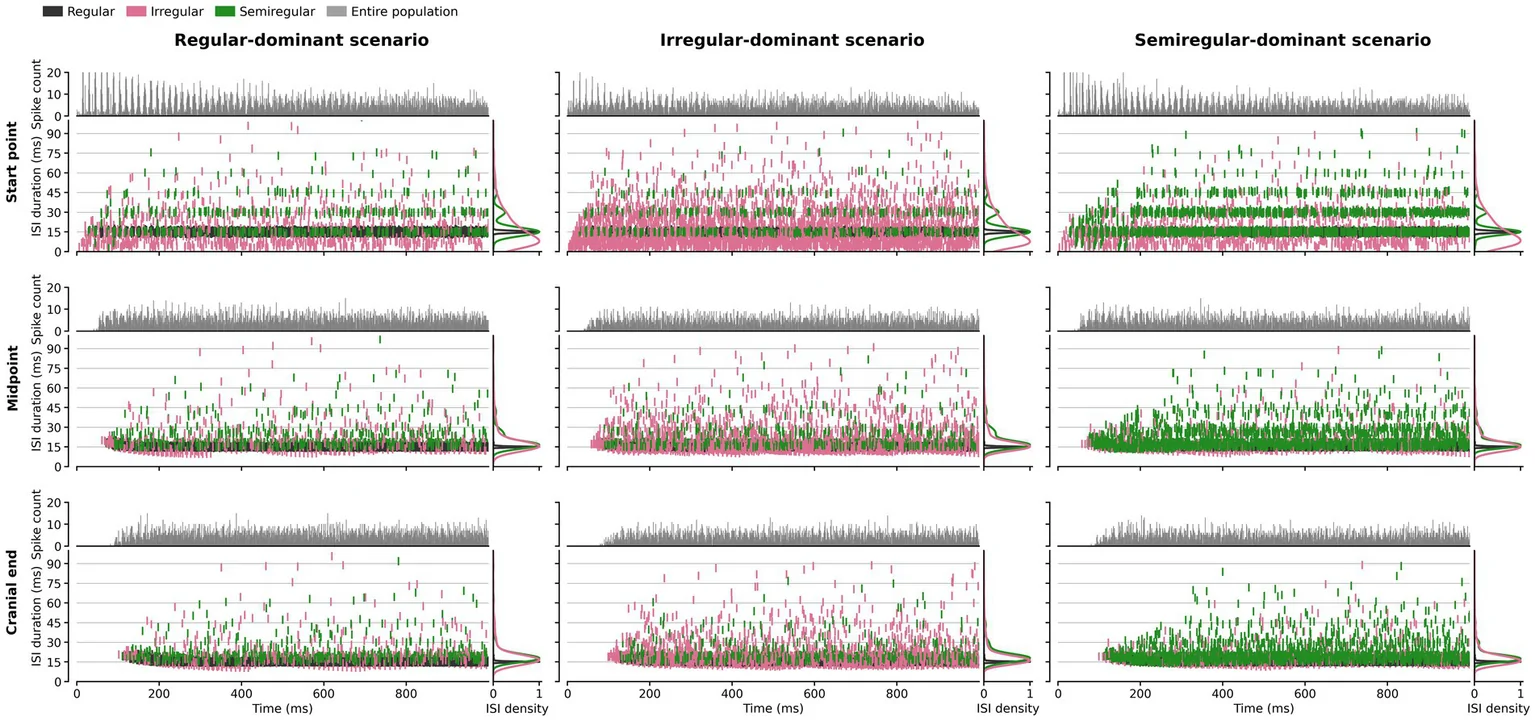
Spike propagation dynamics under heterogeneous diameter conditions ( N=100 , d=2 –3 μm). Diameter heterogeneity disrupted temporal synchronization at the population level, as histogram peaks failed to sharpen during propagation, in contrast to the homogeneous baseline (see Figure 2). Meanwhile, as in the homogeneous baseline, the ISI distributions of semiregular and irregular patterns converged during propagation, whereas regular firing maintained the narrowest range.

Compared to the d=1 μm baseline condition (Figure 2), spike propagation along heterogeneous axons was faster due to their larger diameter ( d=2−3 μm; Figure 11). However, despite faster propagation, diameter heterogeneity strongly disrupted the temporal synchronization of spikes during propagation (Supplementary Table S6). Specifically, spike histogram peaks did not become more pronounced toward the midpoint and cranial end – even in the regular-dominant scenario.

**Figure 12:**
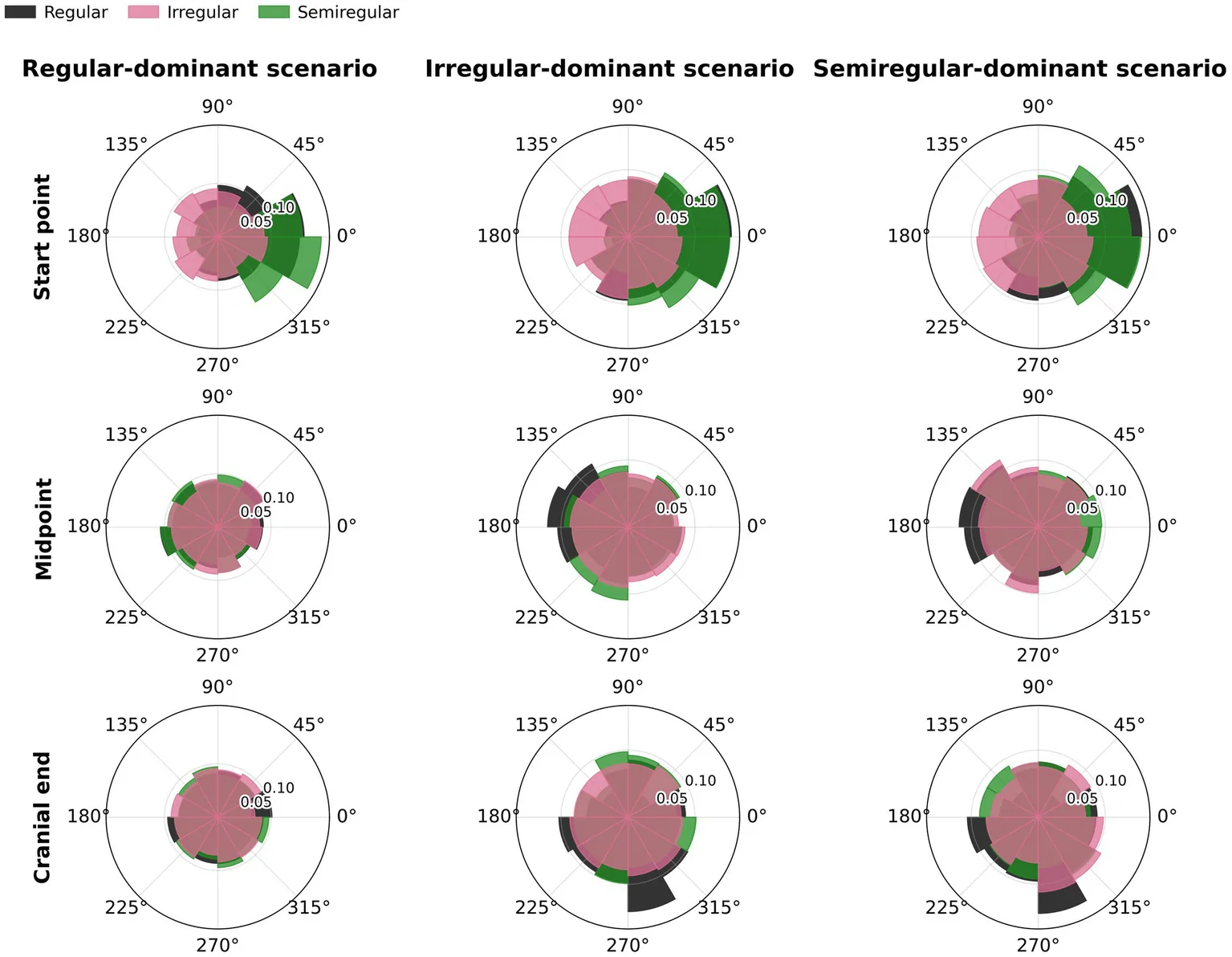
Phase organization of individual spike-pattern types under heterogeneous diameter conditions ( N=100 , d=2 –3 μm). Diameter heterogeneity disrupts phase concentration during propagation, including for regular firing, which loses its rhythmic organization along the axonal bundle. See Figure 3 for comparison with the homogeneous baseline ( N=100 , d=1 μm).

Furthermore, under the heterogeneous diameter condition, both regular and semiregular firing showed a rapid and pronounced loss of their rhythmicity (Figure 12; Supplementary Table S7). This contrasts with the homogeneous baseline (Figure 3; Supplementary Table S2), where only semiregular firing lost its phase-concentrated structure during propagation. Meanwhile, irregular firing was similar under homogeneous and heterogeneous conditions, showing stable broad distribution throughout propagation.

**Figure 13:**
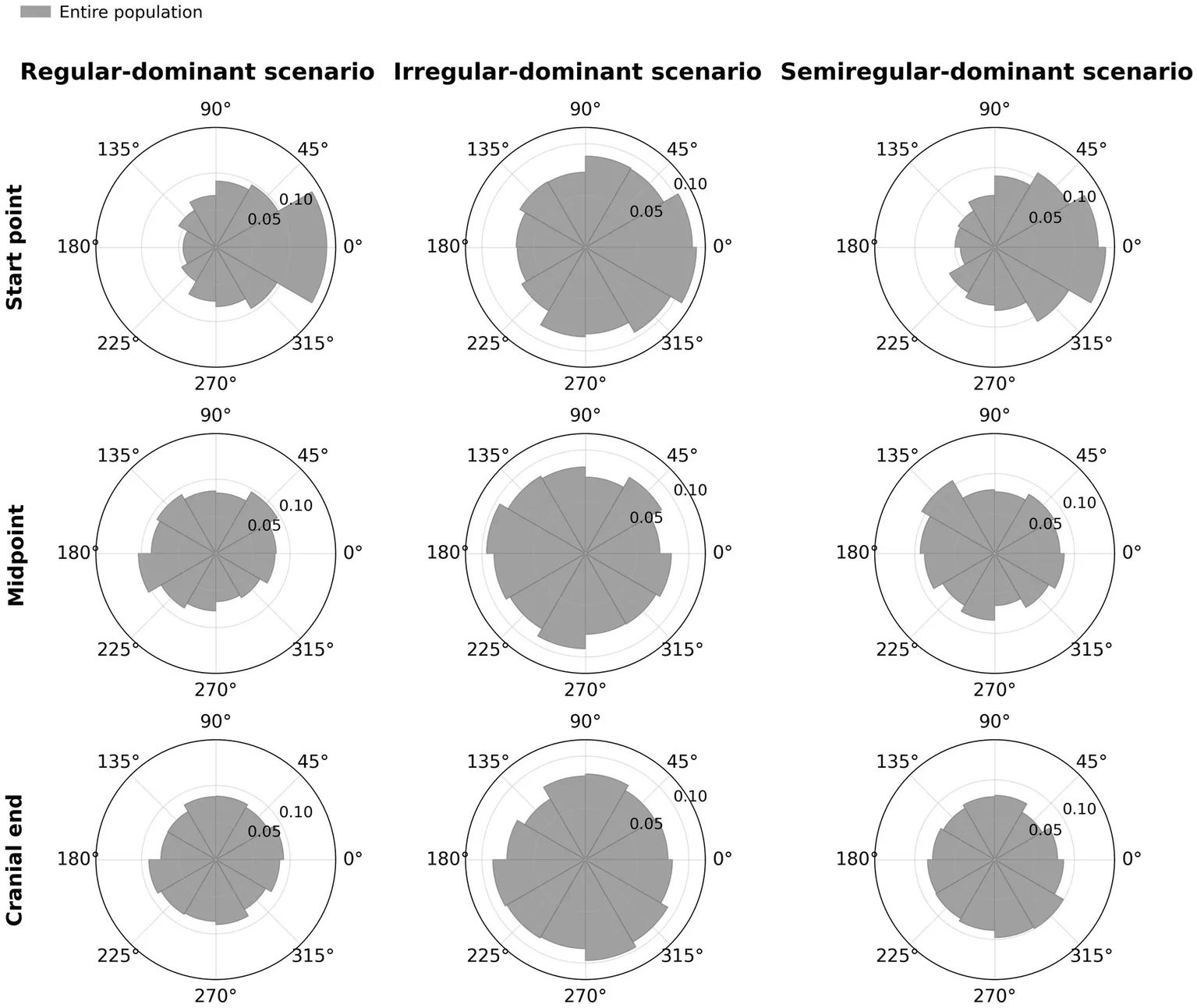
Population-level phase organization under heterogeneous diameter conditions ( N=100 , d=2 –3 μm). Merged phase plots show broadly dispersed population-level phase distributions without a stable preferred phase, indicating that diameter heterogeneity disrupts global rhythmic organization. See Figure 3 for comparison with the homogeneous baseline ( N=100 , d=1 μm).

At the population-level, the heterogeneous bundle did not exhibit stable phase concentration during propagation (Figure 13; Supplementary Table S7). Instead, the merged phase distributions were broadly dispersed and lacked a consistent preferred phase across recording positions. This effect was observed in all three scenarios, even when regular firing dominated. This contrasts with the baseline condition, where population-level rhythmicity was strongest in the regular-dominant scenario (Figure 4; Supplementary Table S2).

Collectively, these findings indicate that while homogeneous architecture preserves the rhythmicity of regular inputs and ephaptic coupling amplifies temporal coherence, diameter heterogeneity disrupts this process by introducing conduction velocity differences that desynchronize even intrinsically rhythmic activity.

## 4 Discussion

A central feature of the present study was the incorporation of physiologically relevant spike-pattern types of LTMRs (Al-Basha and Prescott, 2019) into the ephaptic coupling model (Schmidt and Knösche, 2022). This allowed us to move beyond purely structural determinants of ephaptic synchronization and examine how the temporal structure of afferent activity affects signal propagation. In contrast to the original use of the model, which focused mainly on structural parameters of axonal bundles, here regular, semiregular, and irregular LTMR spike trains were treated as distinct input regimes, interacting through ephaptic coupling during propagation. The main result is that ephaptic coupling promotes partial synchronization of propagating tactile signals in the dorsal column, but this effect is conditional on two factors: the presence of a regular firing component within the afferent population and structural homogeneity of the axonal environment. The regular component serves as a temporal pacemaker, imposing rhythmic order on surrounding axons, whereas diameter homogeneity ensures that this collective rhythmicity is preserved during propagation, allowing ephaptic coupling to synchronize firing across the population.

The pattern-specific responses observed during propagation carry functional implications for tactile signal processing. Our findings demonstrate that the preservation of regular firing’s rhythmicity depends primarily on structural homogeneity of the axonal environment, rather than on ephaptic coupling per se. These results suggest that temporal coding, which relies on precise spike timing to encode stimulus frequency and transients (Mackevicius et al., 2012; Birznieks et al., 2019), benefits from the structural homogeneity of the dorsal column, which preserves the timing of regularly firing LTMR afferents. However, the conversion of this preserved single-fiber rhythmicity into population-wide synchrony requires ephaptic coupling, a mechanism that may be particularly relevant for enhancing the salience of temporal codes (Sagalajev et al., 2024). In contrast, irregular firing remained broadly dispersed across all simulation conditions, suggesting that rate coding, which depends on mean firing rate rather than precise spike timing, requires neither structural homogeneity nor ephaptic coupling to maintain its informational content (Walcher et al., 2018).

Semiregular firing can be conceptualized as a partially degraded version of regular firing, retaining an approximately regular base rhythm but with intermittent missing spikes (Al-Basha and Prescott, 2019). Despite this partial preservation of temporal structure, semiregular trains consistently lost their phase-concentrated organization during propagation across all simulation conditions, whether in homogeneous or heterogeneous environments, with or without ephaptic coupling. This stands in stark contrast to regular firing, which maintained strong phase concentration in homogeneous conditions regardless of ephaptic coupling. This suggests a gating mechanism in the dorsal column, whereby only high-fidelity temporal patterns are preserved to drive population synchronization, while unreliable signals are channeled instead toward rate coding. Temporal coding is inherently more vulnerable to noise, and thus mechanisms must be in place to actively eliminate degraded information while reinforcing what remains intact (London et al., 2010).

We show that ephaptic coupling improves temporal alignment without driving all spikes into full synchrony. This finding aligns with previous computational studies showing that ephaptic entrainment operates in a frequency-dependent manner, where the intensity of synchronization between neural activity and extracellular fields varies with stimulation frequency but does not produce complete phase locking (Cunha et al., 2022). The partial synchronization observed in our simulations also aligns with the view that extracellular field effects operate primarily in the subthreshold range, modulating the timing of near-threshold activity without inducing *de novo* spikes (Anastassiou et al., 2010). Importantly, fully synchronized population responses have been associated with pathological states such as paresthesia and chronic pain, whereas completely disorganized activity impairs coherent tactile perception (Chen et al., 2023; Sagalajev et al., 2024). The partial synchronization observed in our simulations may thus represent an intermediate regime, one that preserves tactile fidelity while avoiding aberrant sensations. This interpretation further suggests a possible mechanism by which additional irregular afferent activity could reduce excessive rhythmic synchronization. For example, in patients with neuropathy, therapy should aim to deliver irregular tactile inputs, thereby increasing temporal dispersion across the neural population. This, in turn, can reduce overly coherent synchronous firing and help alleviate discomfort.

Our findings that structural heterogeneity disrupts the ability of ephaptic coupling to organize spike timing aligns with predictions from the original model (Schmidt and Knösche, 2022), which demonstrated that diameter variability introduces conduction velocity differences that oppose ephaptic synchronization. This is supported by a body of computational work showing that structural parameters critically determine coupling efficacy. Studies have consistently shown that diameter heterogeneity and node misalignment reduce coupling strength (Capllonch-Juan and Sepulveda, 2020; Abdollahi and Prescott, 2024), while fiber geometry and spatial arrangement modulate the probability of synchronization (Binczak et al., 2001; Shneider and Pekker, 2015; Chawla et al., 2021). Here, we extend this insight by showing that heterogeneity not only counteracts synchronization but fundamentally degrades the rhythmic structure of even the most regular inputs. In heterogeneous bundles, regular firing lost its phase-concentrated organization during propagation, whereas in homogeneous environments it retained strong rhythmicity regardless of ephaptic coupling. This suggests that structural uniformity is a prerequisite for preserving spike rhythms in the dorsal column, allowing ephaptic coupling to then entrain the population into partial synchrony. Clinically, this mechanism may have implications for degenerative neuropathies, where reduced structural uniformity within sensory tracts may impair ephaptic synchronization driven by tactile rhythms, thus contributing to sensory deficits (Reutskiy et al., 2003; Naud and Longtin, 2019).

It bears acknowledging, however, that our model carries certain limitations. The simulated axonal bundle is a reduced representation of dorsal column fibers and does not include the full anatomical complexity of the somatosensory pathway. The spike patterns were generated statistically and were not produced by a detailed mechanoreceptor transduction model. In addition, the heterogeneous simulations considered a simplified uniform diameter distribution, whereas real sensory pathways may include more complex diameter distributions, spatial organization, and fiber-type-specific conduction properties. Therefore, the present results should be interpreted as a mechanistic analysis of how spike-pattern structure, ephaptic coupling, and axonal homogeneity interact, rather than as a complete physiological model of tactile perception.

## 5 Conclusion

This study demonstrates that ephaptic coupling in the dorsal column promotes partial synchronization of propagating tactile signals, but this effect critically depends on both the rhythmic structure of afferent input and the structural homogeneity of the axonal environment. Using a biophysically detailed computational model, we showed that regular LTMR firing serves as a temporal pacemaker that imposes rhythmic order on surrounding axons, whereas semiregular and irregular patterns fail to drive population-wide synchrony. Structural homogeneity was essential for preserving this rhythmicity during propagation, whereas diameter heterogeneity disrupted temporal organization even for the most regular inputs. These findings extend previous work on ephaptic coupling by incorporating physiologically relevant spike-pattern diversity alongside structural determinants. Temporal coding is selectively facilitated by ephaptic coupling, but only under structural homogeneity, while rate coding remains independent of both. The partial synchronization observed here may represent an intermediate regime that preserves tactile fidelity while avoiding pathological hypersynchrony, suggesting clinical implications for neuropathies and spinal cord stimulation. Although our model simplifies the anatomical complexity of the somatosensory pathway, it provides a mechanistic framework for understanding how the temporal structure of LTMR activity, ephaptic interactions, and axonal architecture jointly shape the transmission of tactile information to supraspinal targets. Future work incorporating more detailed anatomical features of the dorsal column, along with peripheral encoding and brainstem relay processing, will be necessary to fully elucidate the role of ephaptic coupling in tactile perception and neuromodulation.

## Data Availability

The datasets presented in this study can be found in online repositories. The names of the repository/repositories and accession number(s) can be found: https://gitlab.sirius-web.org/brain/ephaptic-ltmr-patterns.

## References

[ref1] Abdollahi N. Prescott S. A. (2024). Impact of extracellular current flow on action potential propagation in myelinated axons. J. Neurosci. 44:e0569242024. doi: 10.1523/JNEUROSCI.0569-24.2024, 38688722 PMC11211723

[ref2] Abraira V. E. Ginty D. D. (2013). The sensory neurons of touch. Neuron 79, 618–639. doi: 10.1016/J.NEURON.2013.07.051, 23972592 PMC3811145

[ref3] Al-Basha D. Prescott S. A. (2019). Intermittent failure of spike propagation in primary afferent neurons during tactile stimulation. J. Neurosci. 39, 9927–9939. doi: 10.1523/JNEUROSCI.0975-19.2019, 31672792 PMC6978946

[ref4] Anastassiou C. A. Koch C. (2015). Ephaptic coupling to endogenous electric field activity: why bother? Curr. Opin. Neurobiol. 31, 95–103. doi: 10.1016/j.conb.2014.09.002, 25265066

[ref5] Anastassiou C. A. Montgomery S. M. Barahona M. Buzsáki G. Koch C. (2010). The effect of spatially inhomogeneous extracellular electric fields on neurons. J. Neurosci. 30, 1925–1936. doi: 10.1523/JNEUROSCI.3635-09.2010, 20130201 PMC6633973

[ref6] Anastassiou C. A. Perin R. Markram H. Koch C. (2011). Ephaptic coupling of cortical neurons. Nat. Neurosci. 14, 217–223. doi: 10.1038/NN.2727, 21240273

[ref7] Arvanitaki A. (1942). Effects evoked in an axon by the activity of a contiguous one. J. Neurophysiol. 5, 89–108. doi: 10.1152/JN.1942.5.2.89

[ref8] Basser P. J. (1993). Cable equation for a myelinated axon derived from its microstructure. Med. Biol. Eng. Comput. 31, S87–S92. doi: 10.1007/BF02446655, 8231331

[ref9] Binczak S. Eilbeck J. C. Scott A. C. (2001). Ephaptic coupling of myelinated nerve fibers. Phys. D 148, 159–174. doi: 10.1016/S0167-2789(00)00173-1

[ref10] Birznieks I. McIntyre S. Nilsson H. M. Nagi S. S. Macefield V. G. Mahns D. A. . (2019). Tactile sensory channels over-ruled by frequency decoding system that utilizes spike pattern regardless of receptor type. eLife 8:e46510. doi: 10.7554/ELIFE.46510, 31383258 PMC6684274

[ref11] Bokil H. Laaris N. Blinder K. Ennis M. Keller A. (2001). Ephaptic interactions in the mammalian olfactory system. J. Neurosci. 21:RC173. doi: 10.1523/JNEUROSCI.21-20-J0004.2001, 11588203 PMC6763860

[ref12] Buzsáki G. Anastassiou C. A. Koch C. (2012). The origin of extracellular fields and currents — EEG, ECoG, LFP and spikes. Nat. Rev. Neurosci. 13, 407–420. doi: 10.1038/nrn3241, 22595786 PMC4907333

[ref13] Capllonch-Juan M. Sepulveda F. (2020). Modelling the effects of ephaptic coupling on selectivity and response patterns during artificial stimulation of peripheral nerves. PLoS Comput. Biol. 16:e1007826. doi: 10.1371/JOURNAL.PCBI.1007826, 32479499 PMC7263584

[ref14] Chawla A. Morgera S. Snider A. (2021). On axon interaction and its role in neurological networks. IEEE/ACM Trans. Comput. Biol. Bioinform. 18, 790–796. doi: 10.1109/TCBB.2019.2941689 31536014

[ref15] Chen C. Sun L. Adler A. Zhou H. Zhang L. Zhang L. . (2023). Synchronized activity of sensory neurons initiates cortical synchrony in a model of neuropathic pain. Nat. Commun. 14:689. doi: 10.1038/s41467-023-36093-z, 36755026 PMC9908980

[ref16] Cunha G. M. Corso G. Miranda J. G. V. Dos Santos Lima G. Z. (2022). Ephaptic entrainment in hybrid neuronal model. Sci. Rep. 12:1629. doi: 10.1038/S41598-022-05343-3, 35102158 PMC8803837

[ref17] Duval T. Saliani A. Nami H. Nanci A. Stikov N. Leblond H. . (2019). Axons morphometry in the human spinal cord. NeuroImage 185, 119–128. doi: 10.1016/J.NEUROIMAGE.2018.10.033, 30326296

[ref18] Goldman L. Albus J. S. (1968). Computation of impulse conduction in myelinated fibers; theoretical basis of the velocity-diameter relation. Biophys. J. 8, 596–607. doi: 10.1016/S0006-3495(68)86510-5, 5699798 PMC1367402

[ref19] Han K.-S. Chen C. H. Khan M. M. Guo C. Regehr W. G. (2020). Climbing fiber synapses rapidly and transiently inhibit neighboring Purkinje cells via ephaptic coupling. Nat. Neurosci. 23, 1399–1409. doi: 10.1038/s41593-020-0701-z, 32895566 PMC7606706

[ref20] Han K.-S. Guo C. Chen C. H. Witter L. Osorno T. Regehr W. G. (2018). Ephaptic coupling promotes synchronous firing of cerebellar Purkinje cells. Neuron 100, 564–578.e3. doi: 10.1016/j.neuron.2018.09.018, 30293822 PMC7513896

[ref21] Handler A. Ginty D. D. (2021). The mechanosensory neurons of touch and their mechanisms of activation. Nat. Rev. Neurosci. 22, 521–537. doi: 10.1038/s41583-021-00489-x, 34312536 PMC8485761

[ref22] Hodgkin A. L. Huxley A. F. (1952). A quantitative description of membrane current and its application to conduction and excitation in nerve. J. Physiol. 117, 500–544. doi: 10.1113/jphysiol.1952.sp004764, 12991237 PMC1392413

[ref23] Hursh J. B. (1939). Conduction velocity and diameter of nerve fibers. Am. J. Phys. 127, 131–139. doi: 10.1152/AJPLEGACY.1939.127.1.131

[ref24] Jæger K. H. Tveito A. (2026). Extracellular stimulation and ephaptic coupling of neurons in a fully coupled finite element-based extracellular—membrane—intracellular (EMI) model. Front. Comput. Neurosci. 20:1755548. doi: 10.3389/fncom.2026.1755548, 41766775 PMC12935970

[ref25] Jefferys J. G. (1995). Nonsynaptic modulation of neuronal activity in the brain: electric currents and extracellular ions. Physiol. Rev. 75, 689–723. doi: 10.1152/physrev.1995.75.4.689, 7480159

[ref26] Johansson R. S. Vallbo A. B. (1979). Tactile sensibility in the human hand: relative and absolute densities of four types of mechanoreceptive units in glabrous skin. J. Physiol. 286, 283–300. doi: 10.1113/JPHYSIOL.1979.SP012619, 439026 PMC1281571

[ref27] Katz B. Schmitt O. H. (1940). Electric interaction between two adjacent nerve fibres. J. Physiol. 97, 471–488. doi: 10.1113/JPHYSIOL.1940.SP003823, 16995178 PMC1393925

[ref28] Kondrakhin P. Dubrovin S. Kabir M. S. Kolpakov F. Sagalajev B. (2025) Ephaptic Coupling and Regular Firing Optimize Tactile Signal Transmission in the Dorsal Column Proceedings - 7th International Conference Neurotechnologies and Neurointerfaces, CNN 2025 40–43

[ref29] Ladenbauer J. Obermayer K. (2019). Weak electric fields promote resonance in neuronal spiking activity: analytical results from two-compartment cell and network models. PLoS Comput. Biol. 15:e1006974. doi: 10.1371/JOURNAL.PCBI.1006974, 31009455 PMC6476479

[ref30] Lieber J. D. Bensmaia S. J. (2022). The neural basis of tactile texture perception. Curr. Opin. Neurobiol. 76:102621. doi: 10.1016/j.conb.2022.102621, 36027737

[ref31] London M. Roth A. Beeren L. Häusser M. Latham P. E. (2010). Sensitivity to perturbations in vivo implies high noise and suggests rate coding in cortex. Nature 466, 123–127. doi: 10.1038/nature09086, 20596024 PMC2898896

[ref32] Lumpkin E. A. Caterina M. J. (2007). Mechanisms of sensory transduction in the skin. Nature 445, 858–865. doi: 10.1038/NATURE05662, 17314972

[ref33] Mackevicius E. L. Best M. D. Saal H. P. Bensmaia S. J. (2012). Millisecond precision spike timing shapes tactile perception. J. Neurosci. 32, 15309–15317. doi: 10.1523/JNEUROSCI.2161-12.2012, 23115169 PMC3752122

[ref34] Naud R. Longtin A. (2019). Linking demyelination to compound action potential dispersion with a spike-diffuse-spike approach. J. Math. Neurosci. 9:3. doi: 10.1186/S13408-019-0071-6, 31147800 PMC6542900

[ref35] Ramon F. Moore J. W. (1978). Ephaptic transmission in squid giant axons. Am. J. Phys. 234, C162–C169. doi: 10.1152/AJPCELL.1978.234.5.C162, 206154

[ref36] Rebollo B. Telenczuk B. Navarro-Guzman A. Destexhe A. Sanchez-Vives M. V. (2021). Modulation of intercolumnar synchronization by endogenous electric fields in cerebral cortex. Sci. Adv. 7:eabc7772. doi: 10.1126/sciadv.abc7772, 33658192 PMC7929504

[ref37] Reutskiy S. Rossoni E. Tirozzi B. (2003). Conduction in bundles of demyelinated nerve fibers: computer simulation. Biol. Cybern. 89, 439–448. doi: 10.1007/S00422-003-0430-X, 14673655

[ref38] Sagalajev B. Zhang T. Abdollahi N. Yousefpour N. Medlock L. Al-Basha D. . (2024). Absence of paresthesia during high-rate spinal cord stimulation reveals importance of synchrony for sensations evoked by electrical stimulation. Neuron 112, 404–420.e6. doi: 10.1016/j.neuron.2023.10.021 37972595

[ref39] Schmidt H. Knösche T. R. (2019). Action potential propagation and synchronisation in myelinated axons. PLoS Comput. Biol. 15:e1007004. doi: 10.1371/JOURNAL.PCBI.1007004, 31622338 PMC6818808

[ref40] Schmidt H. Knösche T. R. (2022). Modelling the effect of ephaptic coupling on spike propagation in peripheral nerve fibres. Biol. Cybern. 116, 461–473. doi: 10.1007/s00422-022-00934-9, 35538379 PMC9287264

[ref41] Sheheitli H. Jirsa V. K. (2020). A mathematical model of ephaptic interactions in neuronal fiber pathways: could there be more than transmission along the tracts? Netw. Neurosci. 4, 595–610. doi: 10.1162/NETN_A_00134, 32885117 PMC7462434

[ref42] Shifman A. R. Lewis J. E. (2019). ELFENN: a generalized platform for modeling Ephaptic coupling in spiking neuron models. Front. Neuroinform. 13:35. doi: 10.3389/fninf.2019.00035, 31214004 PMC6555196

[ref43] Shneider M. N. Pekker M. (2015). Correlation of action potentials in adjacent neurons. Phys. Biol. 12:066009. doi: 10.1088/1478-3975/12/6/066009 26599368

[ref44] Stacey R. G. Hilbert L. Quail T. (2015). Computational study of synchrony in fields and microclusters of ephaptically coupled neurons. J. Neurophysiol. 113, 3229–3241. doi: 10.1152/jn.00546.2014, 25673735 PMC4440237

[ref45] Striebel J. Habibey R. Wendland D. Gehring H. Podoliak E. Pawlick J. S. . (2025). Reproducible human neural circuits printed with single-cell precision reveal the functional roles of Ephaptic coupling. ACS Nano 19, 38457–38471. doi: 10.1021/acsnano.5c11482, 41139300 PMC12613839

[ref46] Subramanian M. Chiang C.-C. Couturier N. H. Durand D. M. (2022). Theta waves, neural spikes and seizures can propagate by ephaptic coupling in vivo. Exp. Neurol. 354:114109. doi: 10.1016/j.expneurol.2022.114109, 35551899 PMC10214533

[ref47] Walcher J. Ojeda-Alonso J. Haseleu J. Oosthuizen M. K. Rowe A. H. Bennett N. C. . (2018). Specialized mechanoreceptor systems in rodent glabrous skin. J. Physiol. 596, 4995–5016. doi: 10.1113/JP276608, 30132906 PMC6187043

[ref48] Xu Y. Jia Y. Ma J. Hayat T. Alsaedi A. (2018). Collective responses in electrical activities of neurons under field coupling. Sci. Rep. 8:1349. doi: 10.1038/s41598-018-19858-1, 29358677 PMC5778049

