# Supplementary material for: Regular afferent activity and structural homogeneity gate ephaptic synchronization in the dorsal column
Source: Front Hum Neurosci. 2026 Aug 6;20:1896515. doi: 10.3389/fnhum.2026.1896515 (PMC13491656; doi:10.3389/fnhum.2026.1896515)
Supplement: Supplementary file 1 [file Data_Sheet_1.PDF]

Table S1. Quantitative summary of ISI variability and population spike-count clustering under baseline homogeneous conditions.

Scenario	Position	Pattern	Mean ISI (ms)	SD ISI (ms)	CV spike count
Regular-dominant	Start point	Regular	15.001418	0.663597	0.518624
		Irregular	17.981475	15.195214	
		Semiregular	21.336471	11.980237	
	Mid point	Regular	15.303208	1.172448	0.754333
		Irregular	20.709485	10.934948	
		Semiregular	21.374563	8.249432	
	Cranial end	Regular	15.787379	1.910761	0.869213
		Irregular	21.227085	10.113026	
		Semiregular	21.637045	7.051305	
Irregular-dominant	Start point	Regular	14.991393	0.658686	0.418444
		Irregular	17.930129	14.922273	
		Semiregular	21.293610	11.490879	
	Mid point	Regular	15.301018	1.140253	0.485872
		Irregular	20.194327	9.570037	
		Semiregular	21.516601	8.513482	
	Cranial end	Regular	15.769353	1.889627	0.554685
		Irregular	20.640055	8.337867	
		Semiregular	21.791209	7.188867	
Semiregular-dominant	Start point	Regular	14.991372	0.658760	0.521571
		Irregular	17.216596	14.511398	
		Semiregular	21.264943	11.525663	
	Mid point	Regular	15.294763	1.134360	0.559633
		Irregular	19.835654	9.262244	
		Semiregular	21.499192	7.981126	
	Cranial end	Regular	15.766068	1.921854	0.610085
		Irregular	20.255564	8.052799	
		Semiregular	21.681062	6.681243	

Table S2. Quantitative summary of phase concentration under baseline homogeneous conditions.

Scenario	Position	Pattern	Phase concentration
Regular-dominant	Start point	Regular	0.330156
		Irregular	0.016880
		Semiregular	0.358879
		Entire population	0.279581
	Mid point	Regular	0.371312
		Irregular	0.067975
		Semiregular	0.106965
		Entire population	0.265527
	Cranial end	Regular	0.367833
		Irregular	0.048450
		Semiregular	0.068648
		Entire population	0.254802
Irregular-dominant	Start point	Regular	0.317446
		Irregular	0.028301
		Semiregular	0.348855
		Entire population	0.106112
	Mid point	Regular	0.433934
		Irregular	0.019230
		Semiregular	0.114341
		Entire population	0.123095
	Cranial end	Regular	0.471224
		Irregular	0.019481
		Semiregular	0.057973
		Entire population	0.113599
Semiregular-dominant	Start point	Regular	0.317357
		Irregular	0.029026
		Semiregular	0.359714
		Entire population	0.274806
	Mid point	Regular	0.425641
		Irregular	0.040961
		Semiregular	0.117245
		Entire population	0.169832
	Cranial end	Regular	0.454428
		Irregular	0.020806
		Semiregular	0.088648
		Entire population	0.115076

Table S3. Quantitative summary of ISI variability and population spike-count clustering in the full-irregular scenario.

Scenario	Position	Pattern	Mean ISI (ms)	SD ISI (ms)	CV spike count
Full-irregular	Start point	Irregular	17.939951	14.759117	0.393273
	Mid point	Irregular	20.151751	9.047117	0.449510
	Cranial end	Irregular	20.524746	7.806458	0.453626

Table S4. Quantitative summary of ISI variability and population spike-count clustering under conditions without ephaptic coupling.

Scenario	Position	Pattern	Mean ISI (ms)	SD ISI (ms)	CV spike count
Regular-dominant	Start point	Regular	15.001471	0.663455	0.519625
		Irregular	17.981471	15.194872	
		Semiregular	21.336548	11.980173	
	Mid point	Regular	15.315822	1.131878	0.607585
		Irregular	20.651700	10.875166	
		Semiregular	21.398926	8.278582	
	Cranial end	Regular	15.797454	1.864169	0.748580
		Irregular	21.035510	9.974323	
		Semiregular	21.529204	6.852922	
Irregular-dominant	Start point	Regular	14.991424	0.658669	0.417505
		Irregular	17.924716	14.922063	
		Semiregular	21.293652	11.490876	
	Mid point	Regular	15.306129	1.140019	0.444876
		Irregular	20.179795	9.607635	
		Semiregular	21.522746	8.525181	
	Cranial end	Regular	15.778366	1.872829	0.485831
		Irregular	20.624800	8.352138	
		Semiregular	21.797473	7.184087	
Semiregular-dominant	Start point	Regular	14.991424	0.658669	0.521571
		Irregular	17.216601	14.511130	
		Semiregular	21.265006	11.525653	
	Mid point	Regular	15.306129	1.140019	0.478264
		Irregular	19.799769	9.209283	
		Semiregular	21.513315	8.005506	
	Cranial end	Regular	15.778366	1.872829	0.490856
		Irregular	20.214136	8.026301	
		Semiregular	21.697695	6.703283	

Table S5. Quantitative summary of phase concentration under conditions without ephaptic coupling.

Scenario	Position	Pattern	Phase concentration
Regular-dominant	Start point	Regular	0.330254
		Irregular	0.016709
		Semiregular	0.359040
		Entire population	0.279688
	Mid point	Regular	0.381812
		Irregular	0.054994
		Semiregular	0.116892
		Entire population	0.275737
	Cranial end	Regular	0.404972
		Irregular	0.050012
		Semiregular	0.071315
		Entire population	0.283461
Irregular-dominant	Start point	Regular	0.317491
		Irregular	0.028361
		Semiregular	0.348885
		Entire population	0.106071
	Mid point	Regular	0.416353
		Irregular	0.030875
		Semiregular	0.117871
		Entire population	0.126963
	Cranial end	Regular	0.446568
		Irregular	0.023719
		Semiregular	0.060699
		Entire population	0.112655
Semiregular-dominant	Start point	Regular	0.317491
		Irregular	0.029000
		Semiregular	0.359791
		Entire population	0.274894
	Mid point	Regular	0.416353
		Irregular	0.058434
		Semiregular	0.117760
		Entire population	0.170982
	Cranial end	Regular	0.446568
		Irregular	0.029687
		Semiregular	0.086233
		Entire population	0.117929

Table S6. Quantitative summary of ISI variability and population spike-count clustering under heterogeneous diameter conditions.

Scenario	Position	Pattern	Mean ISI (ms)	SD ISI (ms)	CV spike count
Regular-dominant	Start point	Regular	15.001435	0.663578	0.517637
		Irregular	17.515801	14.488394	
		Semiregular	21.061840	11.776844	
	Mid point	Regular	15.096318	0.584573	0.366084
		Irregular	19.618009	11.194518	
		Semiregular	21.120078	9.778949	
	Cranial end	Regular	15.211749	0.849176	0.378004
		Irregular	19.921602	10.434309	
		Semiregular	21.062980	8.655975	
Irregular-dominant	Start point	Regular	14.991394	0.658692	0.418178
		Irregular	17.946401	14.921132	
		Semiregular	21.293606	11.490881	
	Mid point	Regular	15.088904	0.585841	0.410303
		Irregular	19.891572	11.279611	
		Semiregular	21.331425	9.849000	
	Cranial end	Regular	15.205891	0.847951	0.412088
		Irregular	19.986985	10.274355	
		Semiregular	21.378753	9.011621	
Semiregular-dominant	Start point	Regular	14.991386	0.658770	0.517143
		Irregular	16.781332	13.819588	
		Semiregular	20.592695	11.035978	
	Mid point	Regular	15.085157	0.586794	0.422324
		Irregular	18.983445	10.181044	
		Semiregular	20.717024	9.001608	
	Cranial end	Regular	15.202030	0.849171	0.406645
		Irregular	19.050643	9.011732	
		Semiregular	20.726381	8.026214	

Table S7. Quantitative summary of phase concentration under heterogeneous diameter conditions.

Scenario	Position	Pattern	Phase concentration
Regular-dominant	Start point	Regular	0.330177
		Irregular	0.025062
		Semiregular	0.341708
		Entire population	0.273040
	Mid point	Regular	0.062293
		Irregular	0.029054
		Semiregular	0.108518
		Entire population	0.062278
	Cranial end	Regular	0.024219
		Irregular	0.018330
		Semiregular	0.016231
		Entire population	0.016067
Irregular-dominant	Start point	Regular	0.317438
		Irregular	0.028386
		Semiregular	0.348853
		Entire population	0.106129
	Mid point	Regular	0.110688
		Irregular	0.022500
		Semiregular	0.056579
		Entire population	0.042518
	Cranial end	Regular	0.175022
		Irregular	0.015616
		Semiregular	0.031310
		Entire population	0.049744
Semiregular-dominant	Start point	Regular	0.317352
		Irregular	0.052827
		Semiregular	0.320938
		Entire population	0.248111
	Mid point	Regular	0.112449
		Irregular	0.070031
		Semiregular	0.043231
		Entire population	0.045520
	Cranial end	Regular	0.175401
		Irregular	0.087170
		Semiregular	0.019578
		Entire population	0.062317
